# Identification and characterization of QTLs for fruit quality traits in peach through a multi-family approach

**DOI:** 10.1186/s12864-020-06927-x

**Published:** 2020-07-29

**Authors:** Zena J. Rawandoozi, Timothy P. Hartmann, Silvia Carpenedo, Ksenija Gasic, Cassia da Silva Linge, Lichun Cai, Eric Van de Weg, David H. Byrne

**Affiliations:** 1 Department of Horticultural Sciences, Texas A&M University, College Station, TX 77843 USA; 2 Embrapa Clima Temperado, BR-392, km 78, Cx. Postal 403, Pelotas, Rio Grande do Sul 96010-971 Brazil; 3 Department of Agricultural and Environmental Sciences, College of Agriculture, Forestry and Life Sciences, Clemson University, Clemson, SC 29634 USA; 4 Department of Horticulture, Michigan State University, East Lansing, MI 48824 USA; 5 Department of Plant Breeding, Wageningen University & Research, Wageningen, Netherlands

**Keywords:** FlexQTL, Peach QTL, Haplotype, Pedigree-based analysis, Titratable acidity, Soluble solids concentration, Blush

## Abstract

**Background:**

Fruit quality traits have a significant effect on consumer acceptance and subsequently on peach (*Prunus persica* (L.) Batsch) consumption. Determining the genetic bases of key fruit quality traits is essential for the industry to improve fruit quality and increase consumption. Pedigree-based analysis across multiple peach pedigrees can identify the genomic basis of complex traits for direct implementation in marker-assisted selection. This strategy provides breeders with better-informed decisions and improves selection efficiency and, subsequently, saves resources and time.

**Results:**

Phenotypic data of seven F_1_ low to medium chill full-sib families were collected over 2 years at two locations and genotyped using the 9 K SNP Illumina array. One major QTL for fruit blush was found on linkage group 4 (LG4) at 40–46 cM that explained from 20 to 32% of the total phenotypic variance and showed three QTL alleles of different effects. For soluble solids concentration (SSC), one QTL was mapped on LG5 at 60-72 cM and explained from 17 to 39% of the phenotypic variance. A major QTL for titratable acidity (TA) co-localized with the major locus for low-acid fruit (*D*-locus). It was mapped at the proximal end of LG5 and explained 35 to 80% of the phenotypic variance. The new QTL for TA on the distal end of LG5 explained 14 to 22% of the phenotypic variance. This QTL co-localized with the QTL for SSC and affected TA only when the first QTL is homozygous for high acidity (epistasis). Haplotype analyses revealed SNP haplotypes and predictive SNP marker(s) associated with desired QTL alleles.

**Conclusions:**

A multi-family-based QTL discovery approach enhanced the ability to discover a new TA QTL at the distal end of LG5 and validated other QTLs which were reported in previous studies. Haplotype characterization of the mapped QTLs distinguishes this work from the previous QTL studies. Identified predictive SNPs and their original sources will facilitate the selection of parents and/or seedlings that have desired QTL alleles. Our findings will help peach breeders develop new predictive, DNA-based molecular marker tests for routine use in marker-assisted breeding.

## Background

Peach [*Prunus persica* (L.) Batsch] is the third most important temperate fruit crop globally in terms of production [1]. Peach fruit quality traits such as flesh texture, color, sweetness, acidity, and other organoleptic attributes affect consumer preference and consumption [2]. Most of these traits are quantitatively inherited and their genetic control is still unclear [3].

In the last decade, the rate of fresh consumption has decreased from 2.3 to 1.3 kg per capita per year in the U.S. [4]. The lack of consistent quality (poor firmness, lack of flavor, low level of sweetness, and non-ripening fruit) is a main reason consumers do not purchase peaches [5]. The primary reason for poor quality is harvesting at immature stages, a lack of good postharvest handling practices, the need for high yields but not necessarily high quality to make production profitable, and the relative ease for selecting for external versus internal fruit traits. Consumers are willing to pay more for fruits of better quality [6] which is the reason for developing branded fruits that consistently provide high quality fruit [7]. Although much progress was made over the last century in the improvement of fruit size, appearance, and firmness, the improvement of internal quality traits such as sugar content, antioxidant content, and tolerance to internal breakdown has lagged behind [8]. A better understanding of the inheritance of these quality traits will improve breeding efficiency and thereby accelerate the development of new cultivars with improved fruit quality [9]. Also, these traits are complex and are affected by genetics, environment, the interaction between genetics and environment, and cultural practices [5]. When selecting for superior cultivars, it is important to better understand all forces that contribute to the phenotype of the plant, as well as how they interact.

The genetic map construction with quantitative trait loci (QTL) analysis is vital for detecting candidate genes and predictive molecular markers associated with quality traits. In peach, this work has been facilitated by its short juvenile period [10], a simple genome in terms of ploidy level (2x) and size (265 Mb), and the availability of a high-quality reference genome sequence [11]. In the last two decades, abundant genetic maps of important crops have been established including peach [10, 12, 13]. QTLs of SSC have been mapped to linkage groups (LGs) 2–6 [3, 14] and QTLs for organic acids have been mapped to LGs 1, 2, and 4–6 [14, 15]. QTLs associated with chilling injury and maturity date have been reported on multiple LGs with diverse levels of reliability [14]. For some of these QTLs, predictive molecular markers are available and used in breeding [16, 17].

Blush on the skin surface is an important trait that enhances the aesthetic appeal to consumers. In addition, the anthocyanin compounds that create the skin color may have health benefits as a source of antioxidants [18] which may in turn be an element for promoting the peach commercially [2]. Several studies have reported QTLs associated with blush on peach fruits [3, 13, 16] on LG3, LG4, LG5, and LG6 in which LG3 and LG4 were more frequent LGs. The interval on LG3 where the major QTL for blush (*Blush.Pp.ZC-3.1*) is located, contains the candidate genes for skin and flesh coloration of peach (*PprMYB10*), apple (*MdMYB1/MdMYBA/ MdMYB10*), and cherry (*PavMYB10*) [19].

Peach fruits are expected to have a sweet taste, and consumer acceptance is associated with ripe soluble solids concentration reaching 10–12% for high acid and 15–16% for low acid cultivars [20]. Soluble solids concentration (SSC) has low to moderate heritability, which allows for enhancing sugar content even with the environmental, maturity, and production variations [12]. A major SSC QTL was consistently detected in the middle region of LG4 close to the maturity date (MD) locus in intraspecific full-sib families [3]. Minor QTLs have also been reported on LG1, 2, 3, 5, 6, 7, and 8 [14, 21].

Fruit acidity, like SSC, impacts consumer acceptance and is considered a major selection criterion in peach breeding [2, 22]. Low fruit acidity is associated with the *D*-locus located on the proximal end of LG5 [15, 21, 22]. Several additional QTLs with minor effects have been mapped on five other LGs: LG1 and LG6 [15] and LG2, 3, and 7 [21].

Incorporation of molecular tools for peach and fruit-tree breeding is still behind compared to agronomic crop breeding due to several reasons including less funding, a significantly higher investment cost per seedling, and longer juvenility [23].

Although various QTLs have been identified, only a few have been translated into diagnostic DNA tests. This may be attributed to most of the earlier peach QTL mapping studies conducted on single bi-parental populations. Thus, their findings might be limited to particular lineages from the parents of the bi-parental populations [24].

Several DNA tests have been made available to use for several peach traits [25]. DNA test (Ppe-Rf-SSR) which predicts skin color accumulation is available and used for targeting a major *R*_*f*_ locus on LG3 [17]. A DNA test for acidity in peach (CPPCT040) is also available to target the *D*-locus at LG5 [26]. However, peach breeding programs would benefit from developing additional and more predictive DNA tests for acidity, blush, SSC, and other traits to be adopted and used across diverse peach breeding programs.

The main goals of this study are to identify new and validate previously reported QTL(s), to estimate QTL genotypes for important breeding parents, and to identify predictive single SNP(s) associated with desired QTL alleles for three important fruit quality traits: SSC, titratable acidity (TA), and blush through a multi-family approach (pedigree-based analysis) on Texas low to medium chill peach/nectarine germplasm. Results from this work will facilitate the design of DNA tests linked to these QTL(s) or genes to be used for marker-assisted breeding.

## Results

### Phenotypic data analysis

The mean blush value ranged from 2.8 ± 0.78 (CA12) to 3.5 ± 0.91 (TX13) and a maximum range of 4, with number of observations ranged between 62 (TX12) and 143 (overall mean) (Additional file 1: Table S1). The TX and the overall mean were slightly skewed towards high blush ratings whereas the CA blush distribution was slightly skewed towards lower blush ratings (Additional file 2: Fig. S1). SSC exhibited an average between 11.6 ± 1.83 (CA12) and 12.8 ± 2.57 (TX13), with a greater (14.3) and lower (8.1) SSC ranges in TX13 and the overall mean data sets, respectively. All data sets were slightly skewed towards low SSC. TA mean values ranged from 0.6 ± 0.25 (TX12), 0.8 ± 0.37 (CA11) with TA range from 0.9 to 1.4, respectively. The minimum number of observations (43) was recorded for TX12 compared to 137 observations for the overall mean data set. All data sets showed a bimodal distribution, with high acid and low acid group.

No strong correlations were found among the three studied traits (data not shown).

### Genotype by environment interactions

Genotype by environment interaction (G × E) is frequent in multi-environment trials and represents differential responses of genotypes across diverse environments which, if large, selection for the trait cannot be done in only one environment. In this study, TA showed very high broad sense heritability (H^2^ = 0.93), strong correlations among environments (*r* = 0.94), and minimal G × E variance (${\upsigma}_{\mathrm{g}\times \mathrm{e}}^2/{\upsigma}_{\mathrm{g}}^2$ ratio = 0.21) (Additional file 1: Table S2 and S3) whereas the other two traits, blush and SSC, showed high broad sense heritability (H^2^ = 0.81 and 0.76 respectively), strong to moderate correlations among environments (*r* = 0.72 and 0.58 respectively) and a moderate genotype by environment interaction (${\upsigma}_{\mathrm{g}\times \mathrm{e}}^2/{\upsigma}_{\mathrm{g}}^2=$ 0.92 and 1.27, respectively). The higher G × E effect for blush and SSC as compared to TA is further supported by the higher PC2 values (10.5 and 13.5% vs 3.5% respectively) (Additional file 1: Table S4), implying that the environments did discriminate among the populations for blush and SSC. Finally, the minimal G × E effect of TA is supported by the equal distance and similar length of the environmental vectors in the GGE biplots indicating a high correlation among and equal discriminatory ability of the three environments (Additional file 2: Fig. S2).

For blush, the sharper angle and less distance were observed between CA12 and TX12 as compared to CA11 and TX13 indicating a stronger correlation between these environments (*r* = 0.64 vs. 0.30) (Additional file 1: Table S3). In contrast, the best discrimination of blush among genotypes is seen in the CA11 environment indicated by the longer vectors for these environments (Additional file 2: Fig. S2). GGE biplot for SSC showed that CA11 and CA12 were better correlated (*r* = 0.67) as compared to the other environments, with TX13 being a more discriminating environment.

### Genome-wide QTL analysis

The narrow sense heritability (h^2^) varied among datasets in each trait. Minimum h^2^ (0.32) for blush was observed in Blush-CA11 versus maximum observed h^2^ (0.55) in Blush-mean (Table 1). While for SSC, h^2^ ranged from 0.29 (SSC-CA11) to 0.47 (SSC-CA12). Greater range of h^2^ (0.53) was observed in TA compared to blush (0.23) and SSC (0.18) with a minimum (0.33) in TA-TX12 and maximum (0.86) in TA-CA12.

**Table 1:** QTL mapped for the blush, soluble solids concentration (SSC), and titratable acidity (TA) traits evaluated in different environments (CA11, CA12, TX12, TX13), and the overall combined mean for 143 peach seedlings

									***2ln(BF)***		
***Trait***	***MCMC***	***Records***	***μ***	***σ***^***2***^_***p***_	***σ***^***2***^_***e***_	***σ***^***2***^_***A***_	***h***^***2***^	***LG***	***1/0***	***2/1***	***3/2***
Blush-CA11	150,000	103	3.08	0.56	0.38	0.18	0.32	1	2.6	0.6	0.3
Blush-CA12	150,000	138	2.79	0.60	0.29	0.31	0.52	4	13.2	1.1	0.8
								5	2.4	1.8	0.0
								6	3.9	1.0	−0.2
Blush-TX12	150,000	62	3.18	0.62	0.41	0.20	0.33	4	5.7	0.9	0.8
Blush-TX13	150,000	110	3.48	0.83	0.49	0.33	0.40	4	5.1	1.7	1.6
Blush-mean	100,000	143	3.06	0.47	0.21	0.26	0.55	4	16.1	1.6	−0.5
								6	2.0	1.1	−0.9
SSC-CA11	100,000	105	11.87	4.94	3.52	1.42	0.29	5	2.6	0.9	na
SSC-CA12	100,000	137	11.61	3.35	1.79	1.56	0.47	5	13.8	4.0	1.3
SSC-TX13	100,000	111	12.84	6.63	4.59	2.04	0.31	4	2.3	0.4	0.8
								5	9.6	1.0	0.1
SSC-mean	100,000	137	11.90	2.46	1.43	1.03	0.42	4	6.1	0.3	−2.0
								5	11.8	0.9	−0.5
TA-CA11	100,000	95	0.78	0.14	0.03	0.11	0.79	5	7.6	4.2	2.1
TA-CA12	2500,000	131	0.71	0.14	0.02	0.12	0.86	5	11.8	6.0	5.4
TA-TX12	150,000	43	0.55	0.06	0.04	0.02	0.33	5	5.9	0.1	−0.6
TA-mean	500,000	137	0.72	0.13	0.03	0.10	0.77	5	na	6.8	5.6

Blush = blush visually based on % coverage of red blush on skin using 0–5 scale (0 = 0% red coverage, 1 = 1–20%, 2 = 21–50%, 3 = 51–80%, 4 = 81–99%, 5 = 100%); SSC = soluble solids concentration in °Brix; TA = titratable acidity %

CA11 = Fowler, California 2011, CA12 = Fowler, California 2012, TX12 = College Station, Texas 2012, TX13 = College Station, Texas 2013

Markov chain Monte Carlo (MCMC) run length, phenotypic mean (*μ*), phenotypic variance (*σ*^*2*^_*P*_), residual variance(*σ*^*2*^_*e*_), additive variance(*σ*^*2*^_*A*_), narrow-sense heritability (*h*^*2*^), the linkage groups (LG) that QTLs were mapped on

*2ln(BF)*. Bayes Factor, a measure quantifies the support from the data for the number of QTL(s) in the model (QTL evidence), after pair-wise model comparison (1/0, 2/1, and 3/2) such as ‘one-QTL model’ vs. ‘zero-QTL model, etc. *2ln(BF)* < 0 = no evidence; 0–2 = hardly any; 2–5 = positive; 5–10 = strong; > 10 = decisive. Bayes Factor will not be available (na) if either model does not have enough samples in the Markov chain

Although candidate QTLs for blush were identified on four linkage groups (LG1, 4–6) across the four environments and their overall mean, only the QTL located on LG4 passed our pre-defined inclusion threshold, showing strong to decisive evidence in each environment, except for CA11 when it did not give any signal (Additional file 2: Fig. S3). SSC QTLs were identified on two LGs across three environments (except TX12) and their overall mean. Although a minor QTL was mapped on LG4 in TX13 and the overall mean with positive and strong evidence, respectively, only the QTL located at the distal end of LG5 passed our inclusion threshold. It showed consistency across environments and in the overall mean analysis with its reliability supported by trace plot patterns (Additional file 2: Fig. S4). Three candidate QTLs were detected on LG5 for TA: one to three QTLs per environment of which two passed our inclusion criteria. A QTL on the proximal end (*qTA5a*) was common to all three environments examined (TA data was not taken for the 4th environment TX13) and their overall mean. A second QTL on the distal end (*qTA5b*) was environment specific detected only in CA and not in TX (Additional file 2: Fig. S5).

**Fig. 1:**
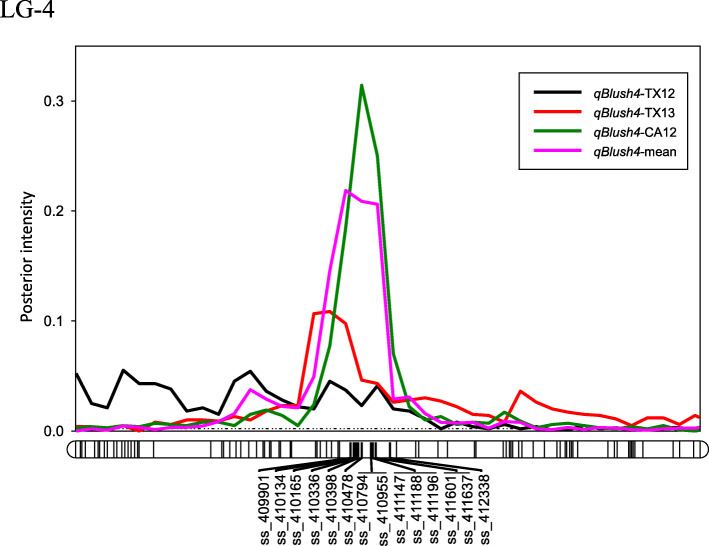
Position of putative QTLs and peaks (large bold font) controlling the blush trait in peach at linkage group 4 (LG4) from four environments (CA11, CA12, TX12, TX13), and the overall combined mean generated using MapChart software [27]. CA11, CA12 = Fowler, California 2011 and 2012; TX12, TX13 = College Station, Texas 2012 and 2013

The proportion of phenotypic variation explained (PVE) by blush QTL on LG4 ranged between 20 and 32%, while the posterior QTL intensity was between 0.24–0.92, and QTL additive effect ranged between 0.53 and 0.63 (Table 2). Peaks for this QTL co-localized across locations and years, having their mode at 42 and 44 cM, and their interval between 40 to 46 cM (Fig. 1) corresponding with the coordinates 10,194,038 to 11,208,347 bp on the peach genome v2.0 [11] (Additional file 1: Table S5 and S6). The PVE by the QTL on LG5 in SSC ranged from 17 to 39% with posterior intensity from 0.27 (CA11) to 0.91 (TX13 and the overall mean) and QTL additive effect between 1.27 (CA12) to 2.32 °Brix (TX13). The peaks of the SSC QTL co-localized across locations and years, having their mode at 60 and 66 cM at the distal end of LG5 and having their interval within the 58 to 72 cM or 14,538,721 to 18,236,497 bp region.

**Table 2:** QTL name, linkage group, interval, mode peak, posterior intensity, additive effect, dominant effect, and phenotypic variance explained (PVE) for the blush, soluble solids concentration (SSC), and titratable acidity (TA) traits evaluated in four environments (CA11, CA12, TX12, TX13), and the overall combined mean for 143 peach seedlings

***QTL name***	***Linkage Group***	***Interval (cM)***	***Mode peak (cM)***	***Posterior intensity***	***Additive Effect***	***Dominant*** ***Effect***	***PVE*** ***(%)***
*qBlush4*-CA12	4	[42, 46]	44	0.92	0.63	–	32
*qBlush4*-TX12	4	[42, 46]	44	0.24	0.62	–	31
*qBlush4*-TX13	4	[40, 46]	42	0.43	0.57	–	20
*qBlush4*-mean	4	[42, 46]	44	0.85	0.53	–	30
*qSSC5*-CA11	5	[58, 72]	66	0.27	1.31	–	17
*qSSC5*-CA12	5	[60, 72]	66	0.90	1.27	–	22
*qSSC5*-TX13	5	[58, 72]	60	0.91	2.32	–	38
*qSSC5*-mean	5	[58, 72]	66	0.91	1.42	–	39
*qTA5a*-CA11	5	[2, 8]	6	0.64	0.33	−0.10	35
*qTA5a*-CA12	5	[2, 8]	6	1.59	0.47	−0.02	74
*qTA5b*-CA12	5	[58, 72]	66	0.68	0.26	−0.10	22
*qTA5*-TX12	5	[2, 8]	4	0.66	0.32	–	72
*qTA5a*-mean	5	[4, 8]	6	0.90	0.49	−0.04	80
*qTA5b*-mean	5	[58, 72]	60	0.70	0.25	−0.13	14

Blush = blush visually based on % coverage of red blush on skin using 0–5 scale (0 = 0% red coverage, 1 = 1–20%, 2 = 21–50%, 3 = 51–80%, 4 = 81–99%, 5 = 100%); SSC = soluble solids concentration in °Brix; TA = titratable acidity %

CA11 = Fowler, California 2011, CA12 = Fowler, California 2012, TX12 = College Station, Texas 2012, TX13 = College Station, Texas 2013

Posterior intensity is the accumulated probability of QTL presence in a successive series of 2 cM bins (chromosome segments) based on Bayesian analysis

For each QTL reported, the evidence [*2ln(BF)*] is either positive (2–5), strong (5–10) or decisive (> 10)

**Fig. 2:**
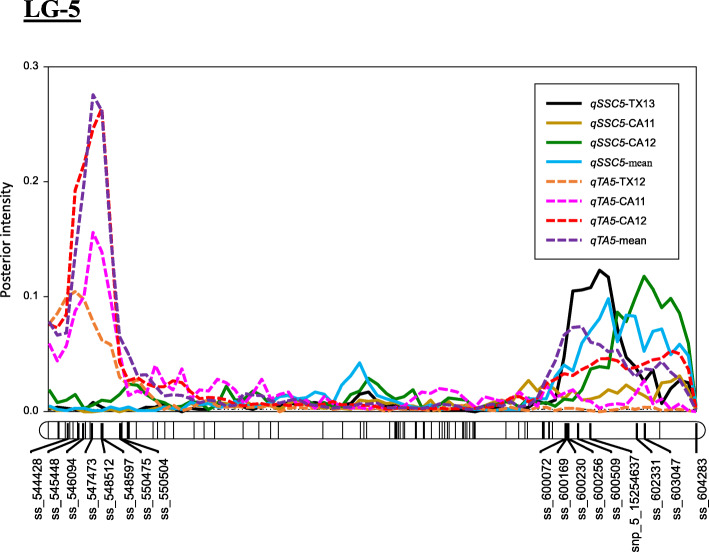
The position of putative QTLs and peaks (large bold font) controlling the soluble solids concentration (SSC), and titratable acidity (TA) for LG5 in peach from four environments (CA11, CA12, TX12, TX13), and the overall combined mean generated using MapChart software [27]. CA11 = Fowler, California 2011, CA12 = Fowler, California 2012, TX12 = College Station, Texas 2012, TX13 = College Station, Texas 2013

The PVE of QTLs for TA on LG5 ranged from 14% (*qTAG5b*-mean) to 80% (*qTAG5a*-mean). The interval of QTL at the proximal part of LG5 ranged from 2 to 8 cM (557,504 – 2,028,804 bp) with a peak at 4 or 6 cM, and from 58 to 72 cM (14,538,721 - 18,236,497 bp) and the peak at either 60 or 66 cM for the QTL at the distal part of LG5 (Fig. 2). The highest posterior QTL intensity (1.59) was associated with *qTA5a*-CA12, and the lowest intensity (0.64) with *qTA5a*-CA11, while the highest value of additive effect (0.49) and negative dominant effects (− 0.13) were recorded for *qTA5a*-mean and *qTA5b*-mean, respectively (Table 2).

### QTL associated haplotypes, number of QTL-alleles, their effect, predictive markers, and sources

A total of 14 SNPs in the predicted *qBlush4* region (42.33–44.83 cM) (Additional file 1: Table S6) chosen for haplotyping revealed four SNP haplotypes across the seven parents in which H1 and H3 were the most prevalent and H2 was the only haplotype associated with high blush (Table 3).

**Table 3:** QTL genotypes for blush, soluble solids concentration (SSC), and titratable acidity (TA) for seven important peach breeding parents, with associated linkage groups, haplotype names, the haplotype’s SNP sequences, and origin sources. QTL alleles for each parent cultivar are presented with ♀ and ♂ for maternal and paternal parent sources, respectively. Parents that are heterozygous for the QTL are in bold. Allele(s) for predictive SNP marker(s) associated with *Q* or *q*-alleles for increasing or decreasing a given trait, respectively, are shown in **underscored bold**. The identity of the SNP markers and their physical and genetic location are given in Additional file 1: Table S6

Trait/LG/Pos.	Parents	QTL allele	Hap.	SNP haplotype	Successive ancestors
				Allele sequence	(founders in bold)
Blush LG4 [42.33–44.83] cM	**Y426–371**	*Q*_*1*_*♀*	H2	**BB**BBBBBBBABBBB	**Y426–371**
	**Y426–371**	*Q*_*2*_*♂*	H3	**BA**AAAAAAABAAAA	**Y426–371**
	**Y434–40**	*Q*_*2*_*♂*	H3	**BA**AAAAAAABAAAA	**Y434–40**
	Galaxy	*Q*_*2*_*♀*	H3	**BA**AAAAAAABAAAA	**Galaxy**
	Y435–246	*Q*_*2*_*♀*	H1	**AB**BBBBBBBABBBB	**Y435–246**
	Y435–246	*Q*_*2*_*♂*	H1	**AB**BBBBBBBABBBB	**Y435–246**
	**Y434–40**	*Q*_*2*_*♀*	H1	**AB**BBBBBBBABBBB	**Y434–40**
	Galaxy	*Q*_*2*_*♂*	H1	**AB**BBBBBBBABBBB	**Galaxy**
	Victor	*q ♀*	H4	**AA**ABABAABAABBB	TropicBeauty > **Fla3–2**
	Victor	*Q*_*2*_*♂*	H1	**AB**BBBBBBBABBBB	Goldprince > **F_Goldprince**
	TX2B136	*Q*_*2*_*♀*	H1	**AB**BBBBBBBABBBB	**TX2B136**
	TX2B136	*Q*_*2*_*♂*	H1	**AB**BBBBBBBABBBB	**TX2B136**
	TXW1490_1	*q ♀*	H4	**AA**ABABAABAABBB	TropicBeauty > **Fla3–2**
	TXW1490_1	*Q*_*2*_*♂*	H1	**AB**BBBBBBBABBBB	**F_TXW1490_1**
SSC LG5 [58.15–72.95] cM	**TX2B136**	*Q ♂*	H6	AAAB**AB**BB	**TX2B136**
	Y435–246	*Q ♀*	H1	BBBA**AB**BB	**Y435–246**
	Y426–371	*Q ♀*	H1	BBBA**AB**BB	**Y426–371**
	Y426–371	*Q ♂*	H1	BBBA**AB**BB	**Y426–371**
	Y434–40	*Q ♀*	H1	BBBA**AB**BB	**Y434–40**
	Y434–40	*Q ♂*	H1	BBBA**AB**BB	**Y434–40**
	Galaxy	*Q ♂*	H1	BBBA**AB**BB	**Galaxy**
	Y435–246	*Q ♂*	H2	BBBA**AB**BA	**Y435–246**
	Victor	*q ♀*	H3	AAABBBAB	TropicBeauty > **Fla3–2**
	**TX2B136**	*q ♀*	H3	AAABBBAB	**TX2B136**
	TXW1490_1	*q ♀*	H3	AAABBBAB	TropicBeauty > **Fla3–2**
	TXW1490_1	*q ♂*	H3	AAABBBAB	**F_TXW1490_1**
	Galaxy	*q ♀*	H4	AAABBBBA	**Galaxy**
	Victor	*q ♂*	H5	BBBAAABA	Goldprince > **F_Goldprince**
Trait/LG/Pos.	Parents	QTL allele	Hap.	SNP haplotype	Successive ancestors
				Allele sequence	(founders in bold)
TA LG5 [2.23–8.12] cM	**Y435–246**	*Q ♂*	H2	**AB**BBBAABBBBB	**Y435–246**
	**Y426–371**	*Q ♂*	H2	**AB**BBBAABBBBB	**Y426–371**
	**Y434–40**	*Q ♂*	H2	**AB**BBBAABBBBB	**Y434–40**
	**Galaxy**	*Q ♂*	H2	**AB**BBBAABBBBB	**Galaxy**
	Victor	*Q ♂*	H2	**AB**BBBAABBBBB	Goldprince > **F_Goldprince**
	Victor	*Q ♀*	H4	**AB**BBBAABBBBA	TropicBeauty > **Flordaprince**
	TX2B136	*Q ♂*	H5	**AB**ABBABBABAB	**TX2B136**
	TX2B136	*Q ♀*	H4	**AB**BBBAABBBBA	**TX2B136**
	TXW1490_1	*Q ♀*	H4	**AB**BBBAABBBBA	TropicBeauty > **Flordaprince**
	TXW1490_1	*Q ♂*	H4	**AB**BBBAABBBBA	**F_TXW1490_1**
	**Y435–246**	*q ♀*	H1	**BA**AAABBAAAAB	**Y435–246**
	**Y426–371**	*q ♀*	H1	**BA**AAABBAAAAB	**Y426–371**
	**Y434–40**	*q ♀*	H3	**BA**ABBAABBBBB	**Y434–40**
	**Galaxy**	*q ♀*	H1	**BA**AAABBAAAAB	**Galaxy**
TA LG5 [58.15–72.95] cM	**TX2B136**	*Q ♂*	H6	**AAABA**BBB	**TX2B136**
	**TX2B136**	*q ♀*	H3	AAABBBAB	**TX2B136**
	Victor	*q ♀*	H3	AAABBBAB	TropicBeauty > **Fla3–2**
	TXW1490_1	*q ♀*	H3	AAABBBAB	TropicBeauty > **Fla3–2**
	TXW1490_1	*q ♂*	H3	AAABBBAB	**F_TXW1490_1**
	Galaxy	*q ♀*	H4	AAABBBBA	**Galaxy**
	Victor	*q ♂*	H5	BBBAAABA	Goldprince > **F_Goldprince**
	Galaxy	*q ♂*	H1	BBBAABBB	**Galaxy**
	Y435–246	*q ♀*	H1	BBBAABBB	**Y435–246**
	Y435–246	*q ♂*	H2	BBBAABBA	**Y435–246**
	Y426–371	*q ♀*	H1	BBBAABBB	**Y426–371**
	Y426–371	*q ♂*	H1	BBBAABBB	**Y426–371**
	Y434–40	*q ♀*	H1	BBBAABBB	**Y434–40**
	Y434–40	*q ♂*	H1	BBBAABBB	**Y434–40**

**Fig. 3:**
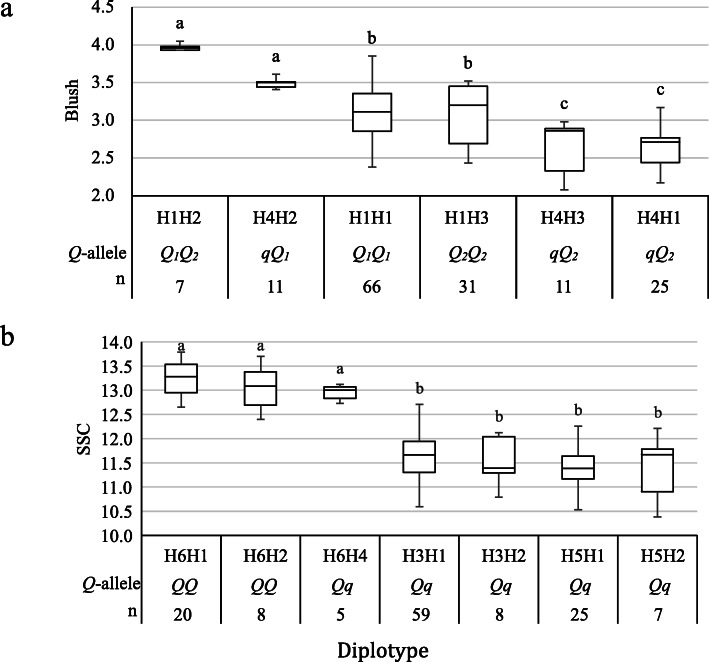
Diplotype effect of the most common haplotypes associated with fruit blush (**a**) and soluble solids concentration (SSC) (**b**) QTLs mapped on peach LG4 and LG5, respectively. Means not connected by the same letter are significantly different (*P < 0.05*) within each linkage group. *n* = Diplotype sample size

The analyses on estimated diplotype effects revealed the presence of three statistically distinct phenotype classes (Fig. 3a). H1 had a greater effect on blush than H4 in the comparisons H1**H1**<>H1**H4** and **H1**H3 <> **H4**H3. Likewise, H2 had a larger effect than H1, H3 and H4 in the comparisons H4**H2**<>H4**H1,** H1**H2** <> H1**H1,** H4**H2**<>H4**H3,** H1**H2** <> H1**H3,** and H1**H2**<>**H4**H1; H3 had a larger effect than H4 (H1**H3** <> **H4**H1). Also, the effects of H1 and H3 could not be differentiated when comparing H1**H1** to H1**H3** and H4**H3** to H4**H1**. The haplotype effects can thus be ordered as H2 > H1 & H3 > H4, thus indicating the presence of three functional QTL alleles with different effects that were coined as *Q*_*1*_, *Q*_*2*_*,* and *q*, respectively.

The four haplotypes could be differentiated from each other by various pairs of SNP markers, like the two adjacent SNP markers [ss_409901 (42.33 cM, 10.5 Mb) and ss_410134 (42.51 cM, 10.6 Mb)], where H2 has the SNP genotype *BB,* H1 *AB,* H3 *BA,* and H4 *AA* (Table 3).

*Q*_*1*_ (H2) was found only in parent Y426–371 and some of its descendants, *Q*_*2*_*’s* H1 is from F_Goldprince, F_TXW1490_1, ‘Galaxy’, TX2B136 and Y435–246, and *Q*_*2*_*’s* H3 is from ‘Galaxy’, Y426–371, and Y434–40, and *q* (H4) is from the selection Fla3–2 through ‘Tropic Beauty’. In this study, ‘Galaxy’, TX2B136, Y426–371, Y435–246, and Y434–40, were considered as founders as their direct parents and earlier generations do not exist or were not available to us for genotyping.

Eight SNPs in the *qSSC5* region (58.15–72.95 cM) were chosen for haplotyping (Additional file 1: Table S6). The results showed six SNP haplotypes across the seven parents, of which H1 and H3 were the most prevalent (Table 3). The analyses of estimated diplotype effects identified two parents as segregating (heterozygous) for the QTL (TX2B136 and ‘Galaxy’) and associated three haplotypes to the *Q*-allele (H1, H2, and H6) and three to the *q*-allele (H3-H5). Diplotype effects analysis was consistent with a bi-allelic QTL (Fig. 3b). The *Q*-allele was associated with an increase of ~ 1.7 °Brix and was associated with the *AB* haplotype of the pair of adjacent SNP markers ss_600256 (14.6 Mb, 58.48 cM) and ss_600509 (14.9 Mb and 59.55 cM).

In the *qTA5a* region (2.23–8.12 cM), 12 SNP markers underwent haplotype analysis and resulted in five SNP haplotypes identified among the seven parents, in which H2 and H4 were the most prevalent (Table 3). FlexQTL indicated that H2, H4, and H5 were associated with high TA, and H1 and H3 with low TA. The observed high intensity for *qTA5a* (1.59) (Table 2) implies that FlexQTL assigned two QTLs to the *qTA5a* QTL interval. The distance between them averaged is just 2.7 cM across all sampled models. This distance is too short to be genetically meaningful with our current population size and might have affected FlexQTL’s QTL genotype assignments. In the second QTL (*qTA5b*) region (58.15–72.95 cM), six SNP haplotypes were identified and only H6 was associated with high TA (Table 3).

Moreover, to distinguish the individual effects of *qTA5a* and *qTA5b*, both QTLs have to be considered simultaneously, e.g. through phenotypic means of their compound genotypes. Therefore, we deviated from our previous analysis workflow by examining QTL-allele – SNP haplotype associations and haplotype effects through a compound diplotype analysis for each family separately (Table 4). The analyses were hampered by the small family sizes, and hence a very low representation of various compound diplotypes. Nevertheless, *qTA5a-*H2 is clearly associated with high TA, and H1 and H3 with low TA. While less information was available for H4 and H5, their effect seemed to be similar to that of H2. Two families (4 and 5) indicated that the effect of H2 was larger at double than at a single dose. Compound diplotypes where H2 occurred together with H4 or H5 showed higher TA values than compound genotypes in which one of these three haplotypes were combined with H1 or H3. Altogether this indicates that *qTA5a*’s H2, H4, and H5 are associated with a *Q*-allele for high acidity and that H1 and H3 are associated with a *q*-allele for low acidity. This outcome of the diplotype analyses was consistent with the *Q/q* allele assignments by FlexQTL. For *qTA5b*, H6 was associated with increased TA values in the presence of a double *Q-*dose at *qTA5a*. The overview on compound *qTA5a*-*qTA5b* diplotypes could be simplified by converting *qTA5’s* diplotypes to QTL genotypes (Additional file 1: Table S7). Our results indicating an epistatic effect of *qTA5a* over *qTA5b*, however, a few compound genotypes carrying both *qTA5b*-*Q* (H6) and *qTA5a*-*QQ* (H2H2, H2H4, or H2H5) did not show increased TA levels (TA > 1.0). This might be due to experimental variation, as the between years variation of a progeny increased with increasing TA levels (Additional file 2: Fig. S6), while a genetic contribution cannot be excluded. With regard to recombination, fewer events occurred in the region of *qTA5a* whereas many recombination events occurred on *qTA5b* but with wide recombination intervals resulting in many recombinant haplotypes for the later one with a frequency of 1.

**Table 4:** Analysis of compound QTL (*qTA5a* and *qTA5b*) diplotypes in seven full-sib peach families for their average titratable acidity (TA) content from the two environments CA11 and CA12. Haplotypes that seemed to be associated with a *Q*-allele for increased TA are in bold. Underlined TA-values are deviating from the proposed genetic model in which *qTA5a* shows recessive inheritance and where expression of *qTA5b* requires *qTA5a* to be*-QQ*

			*qTA5a*									
**FS-Family**		***Diplotype***	***TA***					***Individual count***				
			H3**H4**	H**3H5**	**H2H4**	**H2H5**	***Total***	H3**H4**	H3**H5**	**H2H4**	**H2H5**	***Total***
TX2B136 × Y434–40	*qTA5b*	H1H3	0.42	–	**0.75**	**0.98**	0.71	6	–	4	2	12
		H1H6	0.58	0.60	**1.35**	**1.45**	0.99	2	4	1	5	12
		***Total***	**0.50**	**0.60**	**1.05**	**1.21**	**0.85**	**8**	**4**	**5**	**7**	**24**
		*Conclusions: 1) qTA5a:* Effect H2 > H3, H4 ≡ H5 *2) qTA5b:* Effect H6 > H3*, Effective only in the presence of qTA5a-*H*2*										
			H1**H4**	H1**H5**	**H2H4**	**H2H5**	***Total***	H1**H4**	H1**H5**	**H2H4**	**H2H5**	***Total***
TX2B136 × Y435–246	*qTA5b*	H1H3	0.45	0.40	**0.70**	**–**	0.52	2	1	2	–	5
		H2H3	0.30	0.50	**–**	**1.05**	0.62	1	1	–	1	3
		H2**H6**	0.53	0.20	**0.95**	**–**	0.56	3	1	1	–	5
		***Total***	**0.43**	**0.37**	**0.83**	**1.05**	**0.56**	**6**	**3**	**3**	**1**	**13**
		*Conclusions: 1) qTA5a:* Effect H2 > H1, H4 ≡ H5 *2) qTA5b:* None, too few pairwise comparisons										
			H1**H2**	H1**H4**	**H2H2**	**H2H4**	***Total***	H1**H2**	H1**H4**	**H2H2**	**H2H4**	***Total***
Victor × Y426–371	*qTA5b*	H1H3	0.38	0.35	**0.90**	**0.77**	0.60	3	3	1	3	10
		H1H5	0.43	0.30	**1.08**	**0.97**	0.69	7	2	2	8	19
		***Total***	**0.41**	**0.33**	**0.99**	**0.87**	**0.65**	**10**	**5**	**3**	**11**	**29**
		*Conclusions: 1) qTA5a:* Effect H2 > H1, H2 ≡ H4, H2 at single dose has no effect *2) qTA5b:* H5 possibly slightly > H3 in some genetic backgrounds										
			H1**H2**	H1**H4**	**H2H2**	**H2H4**	***Total***	H1**H2**	H1**H4**	**H2H2**	**H2H4**	***Total***
Victor × Y435–246	*qTA5b*	H1H3	–	0.35	**0.80**	**–**	0.58	–	1	1	–	2
		H1H5	0.35	0.55	**–**	**–**	0.45	1	1	–	–	2
		H2H3	–	–	**–**	**0.85**		–	–	–	2	2
		H2H5	0.40	0.30	**0.85**	**–**	0.52	1	1	4	–	6
		***Total***	**0.38**	**0.40**	**0.83**	**0.85**	**0.51**	**2**	**3**	**5**	**2**	**12**
		*Conclusions: 1) qTA5a:* Effect H2 > H1, H2 at single dose has no effect *2) qTA5b:* None, too few pairwise comparisons										
			*qTA5a*									
**FS-Family**		***Diplotype***	***TA***					***Individual count***				
			H1**H4**	H1**H5**	**H2H4**	**H2H5**	***Total***	H1**H4**	H1**H5**	**H2H4**	**H2H5**	***Total***
TX2B136 × Galaxy	*qTA5b*	H1H3	0.30	0.35	**0.98**	**–**	0.54	1	1	2	–	4
		H1**H6**	0.50	**1.05**	**–**	**1.63**	1.06	1	1	–	3	5
		H4**H6**	0.33	–	**1.00**		0.67	3	–	2		5
		***Total***	**0.38**	**0.70**	**0.99**	**1.63**	**0.76**	**5**	**2**	**4**	**3**	**14**
		*Conclusions: 1) qTA5a:* Effect H2 > H1*,* *2) qTA5b:* None, too few pairwise comparisons H6 > H3										
			H3**H4**		**H2H4**		***Total***	H3**H4**		**H2H4**		***Total***
TXW1490_1 × Y434–40	*qTA5b*	H1H3	0.39		1.03		0.71	4		11		15
		***Total***	**0.39**		**1.03**		**0.71**	**4**		**11**		**15**
		*Conclusion: qTA5a:* Effect H2 > H3										
			H1**H4**		**H2H4**		***Total***	H1**H4**		**H2H4**		***Total***
TXW1490_1 × Y435–246	*qTA5b*	H1H3	0.33		0.75		0.54	2		3		5
		H1**H6**	0.70		0.88		0.79	1		2		3
		***Total***	**0.52**		**0.82**		**0.67**	**1**		**2**		**8**
		*Conclusion*: None, too few data										

Concerning predictive markers for *qTA5a,* each of the two SNP markers can distinguish the *Q* and *q* alleles (ss_544428 at 557,504 bp and ss_544495 at (610,569 bp) (Table 3, Additional file 1: Table S6). Three breeding parents (‘Victor’, TX2B136 and TXW1490–1) were homozygous for the *Q*-allele, while the remaining four parents were heterozygous. The lower TA values were in individuals with diplotypes containing H1 and H3 and were present in Y435–246, Y426–371, Y434–40, and ‘Galaxy’. For *qTA5b*’s, QTL genotypes could be predicted by various pairs of SNP markers that include ss_600509 combined with one of the six markers ss_600072, ss_600169, ss_600230, ss_600256, ss_603047, or ss_604283).

## Discussion

A high percentage of red blush on the fruit surface is desirable for the fresh market peaches and nectarines in the U. S [28]. Blush, a quantitative trait, is expressed during the final stage of fruit development and when the fruit is directly exposed to sunlight [16]. QTLs for blush have been reported on the linkage groups 2–7 [3, 13, 14, 16], indicating the polygenic nature of inheritance.

In this study, the narrow sense heritability of blush was between 0.31 to 0.52 (Table 1), thus falling between previously reported values of 0.19 [29], 0.70 [30], and 0.71 [14]. Heritability is germplasm and environment-specific thus different h^2^ values may be expected among studies [31]. The low (0.29) to moderate (0.47) h^2^ for SSC, which agrees with previous reports [12, 30]. In contrast, all data sets exhibited high h^2^ (0.77 to 0.86) for TA (except for TA-TX12), which was similar to that previously reported [32], suggests the proportion of variation in this trait within our population is more attributed to the genetic component than the environmental effects.

The *qBlush4* region (10.2–11.2 Mb) on LG4 which explained 20–32% of the phenotypic variation, is close to the positions of a major blush QTL previously reported on different peach germplasm, like the region around 11.8 Mb for a QTL with PVE ~ 69% in the family ‘Venus’ × ‘Big Top’ [33], or the 11.2–14.1 Mb region in a multi-parent population [14]. Also, two minor QTLs for blush on LG4 have been reported for the 3.5–4.4 Mb and 7.5–8.8 Mb region in an F_2_ family from a ‘Zin Dai’ × ‘Crimson Lady’ progeny [34] (Additional file 1: Table S8) that had a major QTL on LG3 with a PVE of up to 84%. The QTL resulted from an interval mapping approach, where the minor ones were not validated through a co-factor analysis. The mapped QTL in our study could be the same as these previously reported major QTLs, whereby the variation in QTL positions could be due to the differences in genetic background, differences in mapping methods, or coincidental variation in phenotypic distributions.

In summary, among the four QTLs detected for blush, the QTL on LG4 was more stable and consistently identified in the same chromosome region. In contrast, the three QTLs detected on LG1, LG5, and LG6 appeared environment-specific and expressed only in California (CA11 and CA12). These QTLs resulted in a G × E interaction that observed for blush in which CA11 was a more discriminatory environment compared to others. Generally, fruit in TX had more blush on the skin than CA (3.4 vs. 2.9, data not shown). This quantitative trait is affected by exposure to sunlight and other environmental factors [35, 36]. This interaction could be due to the differences in agricultural practices (e.g. pruning, picking fruit from the inner side of the tree) or weather conditions (e.g. number of cloudy and foggy days), that might prevent exposure to sunlight, and thereby decrease blush development [37]. Less exposure to sunlight would depress the activity of the light-inducible MYB gene-regulating anthocyanin biosynthesis pathway [38]. Also, CA11 data was taken from 2nd leaf trees (2nd growth season) which were very vigorous, and this may explain the discriminatory effect among other environments. More work needs to be done to determine the reason and importance of this interaction.

Examination of the relative effects of haplotypes and estimated QTL genotypes revealed, for the first time, a series of QTL alleles of different effects that we coined *Q*_*1*_*, Q*_*2*_, and *q*. *Q*_*1*_ had the largest effect and was present in just one parent (Y426–371), and the *q* allele for low blush was present in two parents and inherited in both cases from a single source **Fla3–2.** These findings underline the narrow genetic base of our germplasm for high and low blush. *Q*_*2*_ had a weaker effect and was present in each of our parents, underlining its general occurrence in the Texas A&M University breeding program. The use of multi-parent populations for finding multiple functional alleles of different effect was also reported for two acidity QTLs/genes in apple by [39].

The interval of *qBlush4* co-localizes with a major QTL for RD around the markers ss_410398 (10.7 Mb) [40] and ss_411147 (10.9 Mb) [41] (Fig. 1). Also, the moderate correlation between blush and RD estimated in this study (*r* = − 0.42, Additional file 2: Fig. S7) and other studies (*r* = − 0.57 [30], − 0.24 and − 0.56 [3]) may be explained by either the presence of a single QTL with pleiotropic effects or by the linkage between separate QTLs for these traits [15].

More insight in the inheritance may be gained in the future through a multi-parent study in which the known major QTLs are segregating and which is of sufficient size to allow the good representation of the various compound QTL genotypes.

In this study, we mapped a QTL associated with SSC at the distal end of LG5 between ss_600072 and ss_604283 corresponding to the 14.5–18.2 Mb or 58–72 cM intervals, and which exhibited a PVE from 17 to 39% (Table 2 and Additional file 1: Table S5). The interval overlapped with the QTL reported previously [14, 34], and might be different from a QTL reported by [42] that had its peak around the SNP markers ss_572589 and ss_585182 located at 5.8 and 9.2 Mb with a PVE between 13 to 17% (Additional file 1: Table S8). The mapped QTL of this study also overlapped with *G*-locus for controlling fruit type (pubescence vs. glabrous) at the distal end of LG5, spanned from 15.1 to 16.3 Mb on the peach genome [43]. Haplotype analysis revealed that the H6 had a greater effect than other haplotypes on increasing SSC in peaches and was inherited from TX2B136. Furthermore, a minor QTL (SSC-TX13 and SSC-mean) was mapped on LG4 and located between ss_410794 and ss_414387 (43.56–48.43 cM, 10.8–12.1 Mb). Overall, the QTL on LG5 showed more stability as it was mapped consistently across environments and the overall analysis in the same genomic region. While the minor QTL on LG4 was environment-specific as it was only mapped in TX13. As mentioned earlier, TX13 was the more discriminating environment for SSC, indicating that G × E interaction was present for this trait. In general, fruit in TX13 had more SSC than both CA data sets (12.6 vs. 11.5, data not shown) likely due to greater environmental stresses affecting this site such as shallow and droughty soils as well as smaller fruit sizes all of which can result in higher SSC. This trait is strongly influenced by numerous environmental factors including temperature, canopy position, water availability, crop load, and agricultural practices during the fruit development period [37]. No QTL was detected for TX12, probably because of a low number of records in this dataset (*n* = 53).

The first TA QTL (*qTA5a*) at the upper part of LG5, showed recessive inheritance for high acidity and had PVEs between 35 and 80%, indicating this locus had a high contribution to the observed trait variation (Table 2 and Fig. 1). Our findings are consistent with the literature, as *qTA5a* co-localizes with the *D*-locus for fruit acidity in peaches [22], explained 60–87% of the phenotypic variance [15, 32, 42] (Additional file 1: Table S8), and was generally considered to be dominant for low acidity [22].

Our data did not allow adequate estimation of dominance levels for the two TA QTLs as one of the three QTL genotypes was lacking in our study population (*qTA5a-qq*, and *qTA5b-QQ*). In the absence of *qTA5a-qq,* FlexQTL’s dominance estimates are calculated under the assumption that *qq* progeny would not have any TA. The true level of negative dominance is likely to be higher as individuals probably have some base level of TA > 0. The *qTA5a* region has been frequently associated with TA with high PVEs indicating that the *D*-locus has a major effect across a wide range of environments. From a breeding viewpoint, dominance is useful when the dominant allele is directed towards the desired trait level. A single *Q*-dose is sufficient for a relatively large effect which means less need for homozygosity, making breeding goals easier to achieve while at the same time giving flexibility to bring in other traits through the 2nd homolog. However, dominance complicates breeding when it is directed to the less desired trait level. The Texas A&M University breeding program aims at a range of acidity levels, which is reflected by the *qTA5a* genotypes of the seven parents: some were *QQ* (*dd*) for high acidity, others were *Qq* (*dD*) for low acidity but with the potential to raise acidic progenies, and none were *qq* (*DD*) for low acidity.

The new, second QTL for TA, *qTA5b*, mapped at the lower part of LG5 between ss_600072 and ss_604283 within the chromosomal positions between 14.5–18.2 Mb which was not reported previously. It explained 14–22% of the phenotypic variance, was only detected in CA datasets and segregating in ‘TX2B136’ families (Tables 2 and 3). CA11 had lower statistical power for the presence of the second QTL compared to CA12 which may be attributed to the low number of phenotypic data (95 vs.131 records) especially for those progeny that had H*6* (*Q-*allele) (8 vs. 14 progeny) of increasing TA. Hence, averages over years were used to reduce the experimental error and obtain more progeny with phenotypic data.

Also, the fact that this QTL was only mapped in CA could suggest that fruits were picked at a less mature stage (firmer state) which contain higher levels of TA compared to TX. The temperature could also be another factor as CA had cooler temperatures (15 °C) during fruit development (average of the daily maximum and minimum of March and April) compared to TX (20 °C). This QTL has not been previously reported.

The QTL for SSC discovered in this study co-localized with the new TA QTL (*qTA5b*). Both QTLs had the parent TX2B136 as the source for their *Q*-allele, and both were in coupling phase with each other. The co-localization between *qSSC5* and *qTA5b* may indicate that there is a single QTL with pleiotropic effects rather than two functionally independent but genetically linked QTLs. The SNP haplotypes of this novel QTL could be converted into a universal DNA test for both TA ans SSC. Ultimately, the DNA test will be confirmed and deployed in marker-assisted selection tools through marker-assisted parent selection and marker-assisted seedling selection forms. Thus, our results will help Texas peach breeders to make more informed decisions on efficient cross combinations to save time and resources.

Several candidate genes with functional annotation have been identified within the intervals of the main QTLs linked to these traits [44]. On LG4 (10,582,092 to 11,208,347), Prupe.4G185800 gene is described as WD repeat-containing protein and reported to be associated with regulation of the anthocyanin biosynthetic pathway in peach [45], Prupe.4G187100 is also involved in anthocyanin biosynthesis in fruit [46]. NAC072 (Prupe.4G816800) is the candidate gene for maturity date in peach [47]. While four candidate genes were present on LG5 (14,538,721 to 18,236,497) where SSC and TA traits cluster. Prupe.5G241700 gene is described as sucrose synthase 6 is involved with the sugar accumulation process. This enzyme has a low activity at the early stages of fruit development, followed by rapid increase in activity during fruit maturation [48]. The Prupe.5G175100 gene is also present in this region, with functional annotation to probable polygalacturonase and associated with the peach softening. The association between sugar accumulation and softening processes in fruit development has been reported by [49]. Lastly, Prupe.5G172400 (MYB98) associated with the responsible genes for pollen tube guidance and formation of filiform apparatus in *A. thaliana* [50], whereas Prupe.5G208500 (MADS6) has been associated with determining floral organ and meristem identities in rice [51].

These two genes along with probable polygalacturonase were associated with both SSC and ripe date (RD) phenotypes, and the same was identified between the genes associated with both RD and blush on LG4. Thus, our findings suggest that SSC and blush are influenced by RD. A pleiotropic effect of the RD has been reported on several quality traits [3, 14, 40, 47]. In the *D-*locus region for fruit acidity in *Prunus*, several genes have been identified [52–54] and none of them was associated with acidity. Prupe.5G004300 is the only candidate gene previously reported associated with fruit acidity [55].

### Limitations of this study

The low number of FS families combined with the small family sizes that resulted in the lacking/under-representation of compound QTL genotypes, hampered final conclusions on the haplotype effects of the interplay between the two TA QTLs: *qTA5a* and *qTA5b*. The other limitation lays in the lack of genotyped pedigrees for most of our parents, making progenies from different families difficult to link genetically through the identity by descent concept. This reduces the power of QTL discovery and consistent assignment of *Q/q*-alleles.

To overcome limitations of this research, a larger total population size is needed to allow larger representation of QTL genotype classes for estimating QTL effects in case of the presence of G × G interaction and/or multiple QTL alleles at a locus. Additional QTL mapping across a wider range of breeding germplasm is also crucial to validate the QTLs of this study and those reported in the literature in numerous genetic backgrounds. Such research would enhance the estimation of haplotype effects and assigned QTL genotypes along with the original sources of the desired *Q*-alleles of the traits of interest. Fine-mapping and/or the candidate gene (CG) approach should be used in future studies to develop markers useful for MAS.

## Conclusions

Pedigree-Based Analysis successfully detected the location of QTLs associated with blush, SSC, and TA among low-medium chill peach/nectarine germplasm. This technique allows the use of multiple segregating full-sib families with a diverse genetic background to enhance the ability to identify both major and minor QTLs that are associated with quality traits. The minor QTLs of blush and SSC were only seen in specific environments and resulted in a moderate G × E interaction in these traits. Our analysis detected a blush associated QTL at the central part of LG4 which agreed with previous studies [14, 16]. This genomic region was associated with RD in this study and supported by other research [34, 40, 41]. The proximal end of LG5 was related to TA and co-localized with the major locus for low-acid fruit (*D*-locus). A second and new TA QTL mapped at the distal end of LG5 was also associated with SSC and was not reported previously. And this QTL could be only relevant to the TX peach and nectarine breeding program. Haplotype characterization of these major QTLs distinguishes this work over the other QTL studies. Haplotype analysis revealed predominant SNP haplotypes associated with increasing or decreasing levels of each trait. We were able to identify haplotypes and predictive SNPs for desired QTL alleles and their original sources. Moreover, multiple functional alleles of different effects were present in our germplasm for blush. The employment of these predictive SNPs to develop DNA tests will facilitate the selection of parents that have desired haplotypes for population development and in seedling selection to discard undesired seedlings as small plants before planting into the field.

## Methods

### Plant materials

**Fig. 4:**
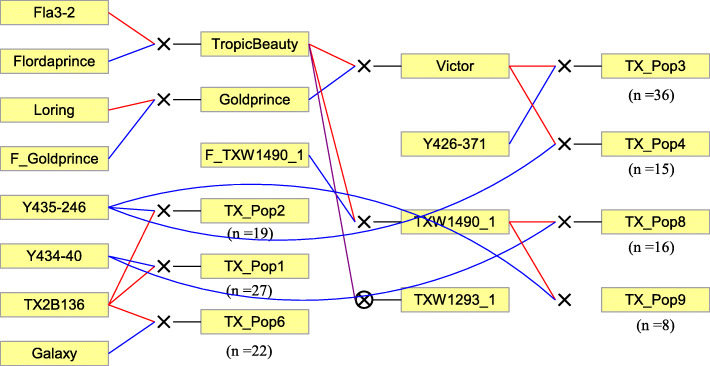
Pedigree of the seven peach families and their progeny number. Red and blue lines link progeny to female and male parents, respectively

This study included 162 seedlings from seven related F_1_ families derived from seven parents descending from 12 founders (Fig. 4). Parents were medium to low chill selections (low-chill and medium-chill require < 300 and 300 to 550 chilling units to release from endodormancy, respectively) from the Texas A&M University breeding program, and medium- to high-chill selections (high-chill requires > 550 chilling unit) from the USDA Stone Fruit Breeding Program in Parlier, CA. The number of seedlings in each family ranged from 8 to 36 with an average of 20. These seedlings, along with parental genotypes, were budded onto ‘Nemaguard’ peach rootstocks and planted in College Station, TX (30°37′41.60″N, 96°22′27.38″W), and Fowler, CA (36°38′21.37″N, 119°42′20.51″W). Each site included one replicate of each seedling and three (Fowler) to four (College Station) replicates of each parent. Phenotypic and fruit quality characteristics of the eight parents used in the study are shown in Additional file 1: Table S9.

### Plot establishment and design

The College Station plot was randomized whereas the Fowler plot was organized by progeny. Trees at College Station were planted in 2010 in staggered double-rows, with 1.7 m between rows, 0.67 m within rows, and 5 m between double rows. All trees were trained as a central leader. Trees at Fowler were planted in 2010, with 4 m between rows, and one meter within rows and trained as a two-scaffold ‘Y’. At each location, irrigation, fertilization, pest and weed control, pruning, and fruit thinning were done according to typical commercial practice.

College Station is located in east central Texas with a sub-humid and warm temperate climate with mild winters and warm to hot, humid summers. Fowler is located in the San Joaquin Valley in central California and is ideal for peach production with a semi-arid Mediterranean climate. The minimum average January temperature and the maximum average July temperature was 4.0 °C and 36.5 °C for Fowler and 7.0 °C to 35.0 °C for College Station, respectively. College Station has greater rainfall than Fowler (1022 versus 248 mm), higher humidity (67.5% versus 55.1%), warmer night temperatures during fruit development (15.8 °C versus 12.4 °C), and more cloudy days (College Station receives 27% less sunlight per year) [56]. In addition, College Station is more subject to late spring freezes, low chill accumulation and has a heavy textured soil. These environmental factors make College Station much less suitable for stone fruit production as compared to Fowler.

### Phenotypic evaluations

All seedlings and parents were evaluated over 2 years (Fowler CA for 2011 and 2012, and College Station, TX for 2012 and 2013) for blush, SSC, and TA. TA was not taken in Texas for 2013. All phenotypic data for the three traits were organized into 11 data sets for QTL analysis. Additionally, the average of all environments was calculated and included as an overall mean dataset for each trait. Descriptive statistics including the mean, maximum, minimum, standard deviation, and the number of observations (records) per environment per trait were obtained from FlexQTL output. When fruits reached the physiological maturity (manually and visually assessment of firmness and background skin color), samples of five fruits were placed in either paper or plastic bags and stored at 1–4 °C for later evaluation.

Subjective scales were used to evaluate fruit blush on each of the five fruits (0–5 scale, 0 = 0% red coverage, 1 = 1–20%, 2 = 21–50%, 3 = 51–80%, 4 = 81–99%, 5 = 100%) as described by Frett [57], and the average was recorded. For biochemical traits, a longitudinal slice of the fruit, approximately 2 cm wide, was taken to extract juice with a juicer for the measurement of SSC from the five-fruits composite sample using a digital refractometer and to measure TA using an automatic titrator (DL 22 Food and Beverage analyzer, Mettler Toledo, Columbus, OH, USA). TA was obtained by the titration of 2 mL peach juice to pH 8.2 with 0.1 N NaOH, expressed as milliequivalents of malic acid, and calculated as:

$$ Titratable\ acidity\ \left(\%\right)=\frac{\Big[ NaOH\ titrated\ (ml)\times 0.1\ N\ (NaOH)\times milliequivalent\ factor\times 100}{6\ g\  of\ juice} $$

with 0.067 as the milliequivalent factor for malic acid [58].

### Broad sense heritability and genotype by environment interaction

A linear mixed model with the residual maximum likelihood (REML) procedure used to estimate the additive (${\sigma}_A^2$), non-additive (${\sigma}_d^2$), and G × E (${\sigma}_{g\times e}^2$) variances for all traits. In the linear mixed model, the genotypes and G × E were considered as random effects and the environments. The results of this mixed model used to quantify the size of the G × E to the genetic variance ratio (${\sigma}_{g\times e}^2/\left({\sigma}_g^2\right)$.

GGE biplots R package version 0.1.1 was used to explain the variation due to genotypes and G × E. The sum of parental [female parent (FP), pollen/male parent (PP)] variances was treated as additive variance (${\sigma}_A^2$), progeny variance was regarded as non-additive variance (${\sigma}_d^2$), and the sum of the parental and progeny variances was regarded as the genotypic variance (${\sigma}_g^2$). The interaction of genotype (FP, PP, or Progeny) by environment was treated as the genetic-environmental variance (${\sigma}_{g\times e}^2$).

Broad sense heritability across the environments was calculated as:

${H}^2=\frac{\sigma_g^2\ }{\sigma_g^2+\frac{\sigma_{g\times e}^2}{E}}$ Where *E* is representing the number of environments [59–61].

Pearson correlations coefficient between phenotypic traits per environment and across environments to give a measure of the strength of linear association using R software.

### SNP genotyping and genetic linkage map

Individuals were previously genotyped as part of the US Peach Crop Reference Set and Breeding Pedigree Set [24] using the IPSC 9 K SNP Array for Peach [62]. The raw iScan data was initially processed into the GenomeStudio software v2010.3 [63] using the Genotyping Module with a Gen Call threshold of 0.15. Parentage records and SNP data curation was performed as described before [64].

After filtering null alleles and non-Mendelian error conflicts across our germplasm 1487 informative SNPs were retained. Their physical position on the peach genome v2.0 [11] was assessed and scaled to an approximate genetic map by using a conversion factor where every 1 Mb corresponded to 4 cM [64]. The markers were evenly distributed over the eight chromosomes.

### QTL detection

Genotypic and phenotypic data for the seedlings were combined for QTL mapping. The pedigree-based QTL analysis approach was implemented through FlexQTL software to increase the accuracy of QTL mapping. It allows for a QTL to be evaluated across diverse genetic backgrounds while, simultaneously, increases the chances of recombination events nearby the QTL of the trait of interest [65, 66]. FlexQTL analyses were conducted on data from each location and the overall mean (of both locations) three times with different chain length, and prior and maximum QTL number to reach an effective chain size (ECS) [67] of at least 100 for phenotypic mean, residual variance and number of QTLs as needed to make sound inferences and conclusions. The length of Markov Chain Monte Carlo (MCMC) simulations varied between 100,000 and 2500,000 iterations, from which one thousand simulations were sampled for statistical inference, thus sampling every 100 to 2500 iterations. ECS values and trace and intensity plots were evaluated for convergence [65]. Traits were first tested with a mixed model (allowing QTLs with additive and dominant effects).

As blush and SSC showed no dominance, they were reanalyzed with an additive model. The statistical evidence for QTLs was evaluated by twice the natural logarithm of the obtained Bayes Factors (BF) [*2ln(BF)*] [68]; values greater than 2, 5 and 10, can be interpreted as indicating positive, strong, and decisive evidence, respectively. For inferences on the number of QTLs, we considered loci that had a 2lnBF ≥ 5, or that 2 ≤ 2lnBF < 5 for at least two data sets. Also, the QTLs with overlapping intervals of at least 2 cM and explained at least 15% of the phenotypic variation were considered for haplotyping. QTL intervals were defined as a series of successive 2-cM bins with intensities corresponding to 2lnBF > 2.

Additive variance (${\sigma}_{A(trt)}^2\Big)$ for each trait was calculated by subtracting the residual variance $\left({\sigma}_e^2\right)$ from the phenotypic variance $\left({\sigma}_P^2\right)$ (both are obtained from FlexQTL). And the narrow sense heritability (h^2^) was calculated as follows:

$$ {h}^2=\frac{\sigma_{A(trt)}^2}{\sigma_P^2} $$

The proportion of phenotypic variance explained (PVE) by each QTL was estimated from FlexQTL output for either additive model (pure additive effect) or mixed model (additive + dominant effects) through one of the following equations:

$$ {\displaystyle \begin{array}{cc}{PVE}_{\mathrm{additive}\kern0.17em \mathrm{model}}=\frac{\sigma_{A\;(qtl)}^2}{\sigma_P^2}\times 100\;\mathrm{where}:& {PVE}_{\mathrm{mixed}\kern0.17em \mathrm{model}}=\frac{\sigma_{A\;(qtl)}^2+{\sigma}_{D\;(qtl)}^2}{\sigma_P^2}\times 100\;\mathrm{where}:\\ {}{\sigma}_{A\;(qtl)}^2:\mathrm{additive}\kern0.17em \mathrm{variance}\kern0.17em \mathrm{of}\;\mathrm{QTL}& \begin{array}{l}{\sigma}_{A\;(qtl)}^2:\mathrm{additive}\kern0.17em \mathrm{variance}\kern0.17em \mathrm{of}\;\mathrm{QTL}\\ {}{\sigma}_{D\;(qtl)}^2:\mathrm{dominant}\kern0.17em \mathrm{variance}\kern0.17em \mathrm{of}\;\mathrm{QTL}\end{array}\end{array}} $$

Our QTL nomenclature is a modification of that of Fan et al. [69]. Thus, in the name *qTTGa*, ‘TT’ stands for the trait, ‘G’ the linkage group number, ‘a’ or ‘b’ letter to distinguish different QTLs for the same trait in one linkage group. Next, an identifier ‘LLYY’ may be added whenever useful to specify the environment where the QTL underlying phenotypic data came from where ‘LL’ specifies the location (State, CA or TX) and ‘YY’ the year in which the trait was evaluated. The QTL name is in italics, while the identifier is not.

### SNP haplotypes and QTL genotypes of important breeding parents

Considering the 1487 informative SNP markers, SNPs within the interval of a significant QTL were chosen for haplotyping. Haplotypes were constructed across the germplasm using FlexQTL and PediHaplotyper [70].

To examine for the presence of multi-allelic QTLs, haplotype effects were analyzed manually. Haplotype effects were deduced from combinations of diplotypes. For instance, the effects of haplotypes H1 and H2 could be estimated by comparing the effects of the H3|H1 and H3|H2 diplotypes. Statistical significance of differences was evaluated using the Steele–Dwass nonparametric multiple comparison test (*P* < 0.05) using JMP Pro Version 13.2 (SAS Institute Inc., Cary, NC, 2016). Then, haplotypes were assigned to QTL allele categories (*Q* or *q*) based on the direction of their effects by increasing or decreasing phenotypic values of each trait. In case of multi-allelic series, *Q* and *q* alleles were differentiated by an index number. Lastly, QTL genotypes were assigned to each individual. The SNP allele sequences of haplotypes along with pedigree records allowed tracing back of favorable alleles to their original sources.

## Supplementary information

**Additional file 1.** Supplemental Tables S1 – S9

**Additional file 2.** Supplemental Figures S1 – S7

## Data Availability

The genotypic and phenotypic datasets of seven full-sib peach families used in this study can be found in the Dryad Repository, doi:10.5061/dryad.tmpg4f4vp (https://datadryad.org/stash/share/oWBiP7isZFdQbY8zS0nTubqrhrT0RntovILSNJp9Xxc).

## Abbreviations

BF: Bayes factor
CG: Candidate gene
cM: Centimorgan
DNA: Deoxyribonucleic acid
ECS: Effective chain size
F_1_: First filial generation
FS: full-sib
h^2^: Narrow-sense heritability
H^2^: Broad-sense heritability
LG: Linkage group
Mb: Megabase pair
MCMC: Markov Chain Monte Carlo
MD: Maturity date
PVE: Phenotypic variance explained
QTL: Quantitative trait loci
SNP: Single nucleotide polymorphism
SSC: Soluble solids concentration
TA: Titratable acidity
