# Supplementary material for: Identification and characterization of QTLs for fruit quality traits in peach through a multi-family approach
Source: BMC Genomics. 2020 Jul 29;21:522. doi: 10.1186/s12864-020-06927-x (PMC7392839; doi:10.1186/s12864-020-06927-x)
Supplement: Supplementary file 1 — Additional file 1. Supplemental Tables S1 – S9 [file 12864_2020_6927_MOESM1_ESM.docx]

| Table S1. Mean, minimum, maximum, standard deviation, and number of observations (N) for blush, soluble solids concentration (SSC), and titratable acidity (TA) in different environments. | | | | | | |
| --- | --- | --- | --- | --- | --- | --- |
| Trait | Environment | Mean | Min | Max | SD | N |
| Blush | CA11 | 3.1 | 2.0 | 5.0 | 0.75 | 103 |
|  | CA12 | 2.8 | 1.0 | 5.0 | 0.78 | 138 |
|  | TX12 | 3.2 | 2.0 | 4.5 | 0.78 | 62 |
|  | TX13 | 3.5 | 1.0 | 5.0 | 0.91 | 110 |
|  | Overall mean | 3.1 | 1.0 | 4.6 | 0.68 | 143 |
|  | | | | | | |
| SSC | CA11 | 11.9 | 7.2 | 20.8 | 2.22 | 105 |
|  | CA12 | 11.6 | 7.6 | 21.1 | 1.83 | 137 |
|  | TX13 | 12.8 | 8.4 | 22.7 | 2.57 | 111 |
|  | Overall mean | 11.9 | 7.6 | 15.7 | 1.57 | 137 |
|  | | | | | | |
| TA | CA11 | 0.8 | 0.3 | 1.7 | 0.37 | 95 |
|  | CA12 | 0.7 | 0.2 | 1.8 | 0.37 | 131 |
|  | TX12 | 0.6 | 0.2 | 1.1 | 0.25 | 43 |
|  | Overall mean | 0.7 | 0.2 | 1.8 | 0.36 | 137 |
| CA11, CA12 = Fowler, California 2011 and 2012; TX12, TX13 = College Station, Texas 2012 and 2013; Overall mean = mean across environments for the trait.  Blush = blush visually based on % coverage of red blush on skin using 0-5 scale (0 = 0% red coverage, 1 = 1%-20%, 2 = 21%-50%, 3 = 51%-80%, 4 = 81%-99%, 5 = 100%); SSC = soluble solids content in º brix; TA = titratable acidity expressed as Eq H^+^/1000 mL of juice. (was not evaluated in TX 2013). | | | | | | |

| Table S2. Estimates of variance components for genotype ($\sigma_{g}^{2}$), genotype × environment ($\sigma_{g\times e}^{2}$), and the ratio of ${\sigma_{g\times e}^{2}}/{\sigma_{g}^{2}}$ and broad sense heritability (H^2^) for blush, SSC, and TA. | | | | |
| --- | --- | --- | --- | --- |
| Trait | $\sigma_{g}^{2}$ | $\sigma_{g\times e}^{2}$ | ${\sigma_{g\times e}^{2}}/{\sigma_{g}^{2}}$ | H^2^ |
| Blush | 0.45 | 0.41 | 0.92 | 0.81 |
| SSC | 1.80 | 2.29 | 1.27 | 0.76 |
| TA | 0.12 | 0.02 | 0.21 | 0.93 |
| Blush = blush visually based on % coverage of red blush on skin using 0-5 scale (0 = 0% red coverage, 1 = 1%-20%, 2 = 21%-50%, 3 = 51%-80%, 4 = 81%-99%, 5 = 100%); SSC = soluble solids content in º Brix; TA = titratable acidity expressed as Eq H^+^/1000 mL of juice. | | | | |

| Table S3. Pairwise correlations among environments for blush, soluble solids concentration (SSC), and titratable acidity (TA). | | | | |
| --- | --- | --- | --- | --- |
| Trait | Environment | by Environment | Correlation coefficient (r) | Number of observations |
| Blush | CA11 | CA12 | 0.42 ^**^ | 103 |
|  | CA11 | TX12 | 0.47 ^**^ | 46 |
|  | CA11 | TX13 | 0.30 ^**^ | 79 |
|  | CA12 | TX12 | 0.64 ^**^ | 59 |
|  | CA12 | TX13 | 0.54 ^**^ | 106 |
|  | TX12 | TX13 | 0.53 ^**^ | 56 |
|  | Among environments | | 0.72 ^**^ |  |
| SSC | CA11 | CA12 | 0.67 ^**^ | 105 |
|  | CA11 | TX13 | 0.41 ^**^ | 82 |
|  | CA12 | TX13 | 0.60 ^**^ | 106 |
|  | Among environments | | 0.58 ^**^ |  |
| TA | CA11 | CA12 | 0.85 ^**^ | 92 |
|  | CA11 | TX12 | 0.62 ^**^ | 31 |
|  | CA12 | TX12 | 0.72 ^**^ | 39 |
|  | Among environments | | 0.94 ^**^ |  |
| ^**^ Pearson correlation is significant at *P≤* *0.01* (2-tailed).  CA11, CA12 = Fowler, California 2011 and 2012; TX12, TX13 = College Station, Texas 2012 and 2013.  Blush = blush visually based on % coverage of red blush on skin using 0-5 scale (0 = 0% red coverage, 1 = 1%-20%, 2 = 21%-50%, 3 = 51%-80%, 4 = 81%-99%, 5 = 100%); SSC = soluble solids content in º brix; TA = titratable acidity expressed as Eq H^+^/1000 mL of juice. (was not evaluated in TX 2013). | | | | |

| Table S4. Trait-wise principal component 1 and 2 variance (PC1 and PC2) of total GGE variation in the traits blush, SSC, and, TA evaluated at Fowler, CA 2011 and 2012 and College Station, TX in 2012 and 2013 environments. | | | |
| --- | --- | --- | --- |
| Traits | GGE | | |
|  | PC1 | PC2 | Sum |
| Blush | 76.0 | 10.5 | 86.5 |
| SSC | 79.4 | 13.5 | 92.9 |
| TA | 95.0 | 3.5 | 98.5 |
| Blush = blush visually based on % coverage of red blush on skin using 0-5 scale (0 = 0% red coverage, 1 = 1%-20%, 2 = 21%-50%, 3 = 51%-80%, 4 = 81%-99%, 5 = 100%); SSC = soluble solids content in º brix; TA = titratable acidity (was not evaluated in TX 2013). | | | |

| Table S5. QTL name, linkage group, along with SNP name, genetic position, and physical location of flanking markers and nearest marker to the center of the mode for the blush, soluble solids concentration (SSC), and titratable acidity (TA) evaluated in four environments (CA11, CA12, TX12, TX13), and the overall combined mean for 143 peach seedlings. | | | | | | | | | | | |
| --- | --- | --- | --- | --- | --- | --- | --- | --- | --- | --- | --- |
|  | | | | ***Flanking markers*** | | | | ***Nearest marker*** | | | |
| ***QTL name*** | ***Linkage***  ***group*** | | | ***Name*** | ***Genetic***  ***position (cM)*** | | ***Physical***  ***location*** | ***Name*** | ***Genetic***  ***position (cM)*** | | ***Physical***  ***location*** |
| *qBlush4*-CA12 | | 4 | ss_409901 | | | 42.33 | 10,582,092 | ss_410794 | | 43.56 | 10,890,653 |
|  | |  | ss_412338 | | | 44.83 | 11,208,347 |  | |  |  |
| *qBlush4*-TX12 | | 4 | ss_409901 | | | 42.33 | 10,582,092 | ss_410794 | | 43.56 | 10,890,653 |
|  | |  | ss_412338 | | | 44.83 | 11,208,347 |  | |  |  |
| *qBlush4*-TX13 | | 4 | ss_408520 | | | 40.78 | 10,194,038 | ss_410165 | | 42.56 | 10,641,209 |
|  | |  | ss_412338 | | | 44.83 | 11,208,347 |  | |  |  |
| *qBlush4*-mean | | 4 | ss_409901 | | | 42.33 | 10,582,092 | ss_410794 | | 43.56 | 10,890,653 |
|  | |  | ss_412338 | | | 44.83 | 11,208,347 |  | |  |  |
| *qSSC5*-CA11 | | 5 | ss_600072 | | | 58.15 | 14,538,721 | ss_602331 | | 66.20 | 16,550,893 |
|  | |  | ss_604283 | | | 72.95 | 18,236,497 |  | |  |  |
| *qSSC5*-CA12 | | 5 | snp_5_15254637 | | | 61 | 15,249,344 | ss_602331 | | 66.20 | 16,550,893 |
|  | |  | ss_604283 | | | 72.95 | 18,236,497 |  | |  |  |
| *qSSC5*-TX13 | | 5 | ss_600072 | | | 58.15 | 14,538,721 | snp_5_15254637 | | 61.00 | 15,249,344 |
|  | |  | ss_604283 | | | 72.95 | 18,236,497 |  | |  |  |
| *qSSC5*-mean | | 5 | ss_600072 | | | 58.15 | 14,538,721 | ss_602331 | | 66.20 | 16,550,893 |
|  | |  | ss_604283 | | | 72.95 | 18,236,497 |  | |  |  |
| qTA5-CA11 | | 5 | ss_544428 | | | 2.23 | 557,504 | ss_548512 | | 6.01 | 1,503,387 |
|  | |  | ss_550475 | | | 8.12 | 2,028,804 |  | |  |  |

| Table S5. *Cont.* | | | | | | | |
| --- | --- | --- | --- | --- | --- | --- | --- |
| *qTA5a*-CA12 | 5 | ss_544428 | 2.23 | 557,504 | ss_548512 | 6.01 | 1,503,387 |
|  |  | ss_550475 | 8.12 | 2,028,804 |  |  |  |
| *qTA5b*-CA12 | 5 | ss_600072 | 58.15 | 14,538,721 | ss_602331 | 66.20 | 16,550,893 |
|  |  | ss_604283 | 72.95 | 18,236,497 |  |  |  |
| qTA5-TX12 | 5 | ss_544428 | 2.23 | 557,504 | ss_546094 | 3.95 | 987,686 |
|  |  | ss_550475 | 8.12 | 2,028,804 |  |  |  |
| *qTA5a*-mean | 5 | ss_546094 | 3.95 | 987,686 | ss_548512 | 6.01 | 1,503,387 |
|  |  | ss_550475 | 8.12 | 2,028,804 |  |  |  |
| *qTA5b*-mean | 5 | ss_600072 | 58.15 | 14,538,721 | ss_600509 | 59.55 | 14,888,402 |
|  |  | ss_604283 | 72.95 | 18,236,497 |  |  |  |
| CA11, CA12 = Fowler, California 2011 and 2012; TX12, TX13 = College Station, Texas 2012 and 2013.  Blush = blush visually based on % coverage of red blush on skin using 0-5 scale (0 = 0% red coverage, 1 = 1%-20%, 2 = 21%-50%, 3 = 51%-80%, 4 = 81%-99%, 5 = 100%); SSC = soluble solids content in º brix; TA = titratable acidity expressed as Eq H^+^/1000 mL of juice. (was not evaluated in TX 2013).  For each QTL reported, the evidence [*2ln(BF)*] is either positive (2-5), strong (5-10) or decisive (>10). | | | | | | | |

| Table S6. SNP name, genetic position (cM), and physical location for SNPs in each allele sequence of haplotypes identified for blush, soluble solids concentration (SSC), and titratable acidity (TA) for seven important peach breeding parents. | | | | | | | | |
| --- | --- | --- | --- | --- | --- | --- | --- | --- |
| **SNP name** | **Genetic position (cM)** | **Physical location** | **Haplotype** | | | | | |
| ***qBlush4*** | | | **H1** | **H2** | **H3** | **H4** |  |  |
| ss_409901 | 42.33 | 10,582,092 | A | B | B | A |  |  |
| ss_410134 | 42.51 | 10,626,874 | B | B | A | A |  |  |
| ss_410165 | 42.56 | 10,641,209 | B | B | A | A |  |  |
| ss_410336 | 42.70 | 10,676,008 | B | B | A | B |  |  |
| ss_410398 | 42.79 | 10,696,489 | B | B | A | A |  |  |
| ss_410478 | 43.04 | 10,760,086 | B | B | A | B |  |  |
| ss_410794 | 43.56 | 10,890,653 | B | B | A | A |  |  |
| ss_410955 | 43.62 | 10,904,526 | B | B | A | A |  |  |
| ss_411147 | 43.69 | 10,921,604 | B | B | A | B |  |  |
| ss_411188 | 43.69 | 10,923,251 | A | A | B | A |  |  |
| ss_411196 | 43.69 | 10,923,464 | B | B | A | A |  |  |
| ss_411601 | 43.91 | 10,976,364 | B | B | A | B |  |  |
| ss_411637 | 43.93 | 10,981,971 | B | B | A | B |  |  |
| ss_412338 | 44.83 | 11,208,347 | B | B | A | B |  |  |
| ***qSSC5*** | | | **H1** | **H2** | **H3** | **H4** | **H5** | **H6** |
| ss_600072 | 58.15 | 14,538,721 | B | B | A | A | B | A |
| ss_600169 | 58.27 | 14,567,044 | B | B | A | A | B | A |
| ss_600230 | 58.44 | 14,610,097 | B | B | A | A | B | A |
| ss_600256 | 58.48 | 14,619,399 | A | A | B | B | A | B |
| ss_600509 | 59.55 | 14,888,402 | A | A | B | B | A | A |
| snp_5_15254637 | 61.00 | 15,249,344 | B | B | B | B | A | B |
| ss_603047 | 67.08 | 16,768,945 | B | B | A | B | B | B |
| ss_604283 | 72.95 | 18,236,497 | B | A | B | A | A | B |

| Table S6. (*Cont.)* | | | | | | | | |
| --- | --- | --- | --- | --- | --- | --- | --- | --- |
| **SNP name** | **Genetic position (cM)** | **Physical location** | **Haplotype** | | | | | |
| ***qTA5a*** | | | **H1** | **H2** | **H3** | **H4** | **H5** |  |
| ss_544428 | 2.23 | 557,504 | B | A | B | A | A |  |
| ss_544495 | 2.44 | 610,569 | A | B | A | B | B |  |
| ss_544961 | 2.79 | 698,215 | A | B | A | B | A |  |
| ss_545261 | 3.29 | 821,356 | A | B | B | B | B |  |
| ss_545448 | 3.40 | 850,261 | A | B | B | B | B |  |
| ss_546094 | 3.95 | 987,686 | B | A | A | A | A |  |
| ss_546316 | 4.20 | 1,049,936 | B | A | A | A | B |  |
| ss_546987 | 4.67 | 1,166,290 | A | B | B | B | B |  |
| ss_547473 | 4.87 | 1,216,762 | A | B | B | B | A |  |
| ss_548512 | 6.01 | 1,503,387 | A | B | B | B | B |  |
| ss_548597 | 6.07 | 1,518,366 | A | B | B | B | A |  |
| ss_550475 | 8.12 | 2,028,804 | B | B | B | A | B |  |
| ***qTA5b*** | | | **H1** | **H2** | **H3** | **H4** | **H5** | **H6** |
| ss_600072 | 58.15 | 14,538,721 | B | B | A | A | B | A |
| ss_600169 | 58.27 | 14,567,044 | B | B | A | A | B | A |
| ss_600230 | 58.44 | 14,610,097 | B | B | A | A | B | A |
| ss_600256 | 58.48 | 14,619,399 | A | A | B | B | A | B |
| ss_600509 | 59.55 | 14,888,402 | A | A | B | B | A | A |
| snp_5_15254637 | 61.00 | 15,249,344 | B | B | B | B | A | B |
| ss_603047 | 67.08 | 16,768,945 | B | B | A | B | B | B |
| ss_604283 | 72.95 | 18,236,497 | B | A | B | A | A | B |

| Table S7. Overview on titratable acidity (TA) content of compound *qTA5a-qTA5b* genotypes across seven full-sib peach families. TA values are averages from the two environments CA11 and CA12. Haplotypes that seemed to be associated with a *Q*-allele for increased TA are in bold. Data came from Table 4 while converting *qTA5a*’s diplotypes to QTL genotypes. For *qTA5a*, our study lacked *qq*-progenies. | | | | | |
| --- | --- | --- | --- | --- | --- |
|  | **Dose *Q*-allele** *qTA5a* | | | | |
| **Diplotype** *qTA5b* | **TA** | |  | **Progeny count** | |
|  | **1** | **2** |  | **1** | **2** |
| H1H3 | 0.37 | 0.85 |  | 24 | 29 |
| H1H5 | 0.41 | 1.03 |  | 11 | 10 |
| H2H3 | 0.40 | 0.95 |  | 2 | 3 |
| H2H5 | 0.35 | 0.85 |  | 2 | 4 |
| Mean | **0.38** | **0.92** | Total | **39** | **46** |
|  |  |  |  |  |  |
| H1**H6** | 0.69 | 1.33 |  | 9 | 11 |
| H2**H6** | 0.37 | 0.95 |  | 4 | 1 |
| H4**H6** | 0.33 | 1.00 |  | 3 | 2 |
| Mean | **0.46** | **1.09** | Total | **16** | **14** |
|  | | | | | |
| Mean | 0.42 | 1.01 | Total | 55 | 60 |

| Table S8. The linkage group and physical position of some previously mapped peach QTLs for blush, soluble solids concentration (SSC), and titratable acidity (TA). | | | |
| --- | --- | --- | --- |
| Trait | LG | Physical position (Mbp) | Source |
| Blush | 3 | 4.8 – 13.9 | [1] |
|  |  | 12.3 – 14.0 | [2] |
|  | 4 | 3.5 – 4.4 | [2] |
|  |  | 7.5 – 8.8 | [2] |
|  |  | 11.2 – 14.1 | [3] |
| SSC | 2 | 19.2 – 20.1 | [3] |
|  |  | 22.9 – 27.1 | [4] |
|  | 4 | 10.1 – 20.2 | [2] |
|  |  | 11.2 – 12.1 | [3] |
|  |  | 14.6 – 25.6 | [2] |
|  |  | 14.7 – 19.2 | [5] |
|  | 5 | 1.0 – 6.0 | [4] |
|  |  | 1.3 – 6.1 | [3] |
|  |  | 5.8 – 9.2 | [6] |
|  |  | 14.6 – 18.2 | [2] |
|  |  | 15.2 – 18.2 | [3] |
|  | 6 | 3.2 – 6.0 | [3] |
|  |  | 7.9 – 12.6 | [4] |
|  |  | 29.2 – 30.1 | [3] |
|  | 7 | 1.1 – 8.3 | [4] |
|  |  | 1.5 – 4.8 | [2] |
|  |  | 16.9 – 19.5 | [2] |
|  |  | 18.1 – 19.2 | [2] |
| TA | 5 | 0.3 – 1.2 | [7] |
|  |  | 0.7 - 1.5 | [6] |
|  |  | 1.0 – 6.0 | [4] |
|  |  | 1.4 – 2.2 | [3] |
|  |  | 12.8 – 13.9 | [6] |
|  | 6 | 7.6.8 – 8.1 | [3] |
|  |  | 23.3 – 26.1 | [3] |
|  |  |  | |

| Table S9. Phenotypic and fruit quality characteristics of the eight parents used in the study. | | | | | | | | |
| --- | --- | --- | --- | --- | --- | --- | --- | --- |
| Genotype | FT | BD | RD | Blush | FW | FD | SSC | TA |
| Y426-371 | Ne-Yel | Feb 18 | May 28 | 90 | 79 | 55 | 12.9 | 0.41 |
| Y434-40 | Ne-Yel | Feb 6 | May 16 | 70-90 | 76 | 55 | 12.7 | 0.44 |
| Y435-246 | Ne-Yel | Feb 22 | Jun 12 | 20-50 | 63 | 50 | 12.5 | 0.34 |
| Galaxy | Pc-Wh | Feb 19 | Jun 12 | 40-70 | 141 | 77 | 12.6 | 0.24 |
| Victor | Pc-Yel | Feb 11 | May 18 | 50-70 | 116 | 64 | 10.7 | 0.87 |
| TX2B136 | Pc-Yel | Feb 5 | Jun 2 | 60-80 | 120 | 63 | 11.0 | 1.29 |
| White Delight Two | Pc-Wh | Feb 20 | Jun 7 | 70-80 | 118 | 62 | 13.0 | 0.33 |
| Tropic Zest Three | Pc-Yel | Feb 5 | Jun 8 | 30-40 | 108 | 62 | 12.2 | 1.00 |
| FT = fruit type (Ne = nectarine, Pc = peach, Yel = yellow fleshed, and Wh = white fleshed); BD = bloom date in Julian days; RD = ripe date in Julian days; Blush = blush visually based on % coverage of red blush on skin; FW = fruit weight in grams; FD = fruit diameter in millimeters; SSC = soluble solids content in ºBrix; TA = titratable acidity expressed as Eq H^+^/1000 mL of juice.  Pantao is heterozygous for round shape; homozygous pantao types do not survive | | | | | | | | |

References

1. Frett TJ, Reighard GL, Okie WR, Gasic K. Mapping quantitative trait loci associated with blush in peach [*Prunus persica* (L.) Batsch]. Tree Genet & Genomes. 2014;10:367-81. doi: 10.1007/s11295-013-0692-y.

2. Frett TJ. Genetic determinism of *Xanthomonas arboricola* pv. *pruni* (Xap) resistance, fruit quality, and phenological traits in peach and incorporation of marker-assisted selection (MAS) in the University of Arkansas peach and nectarine breeding program [Diss.]. Fayetteville, AK: University of Arkansas; 2016. Available from: <https://core.ac.uk/download/pdf/80559387.pdf>.

3. Hernández Mora JR, Micheletti D, Bink M, Van de Weg E, Cantín C, Nazzicari N, et al. Integrated QTL detection for key breeding traits in multiple peach progenies. BMC Genomics. 2017;18:404. doi: 10.1186/s12864-017-3783-6.

4. Fresnedo-Ramírez J, Bink M, van de Weg E, Famula TR, Crisosto CH, Frett TJ, et al. QTL mapping of pomological traits in peach and related species breeding germplasm. Mol Breed. 2015;35:166. doi: 10.1007/s11032-015-0357-7.

5. Dirlewanger E, Cosson P, Renaud C, Monet R, Poëssel JL, Moing A. New detection of QTLs controlling major fruit quality components in peach. Acta Hort. 2006;713:65-72. doi: 10.17660/ActaHortic.2006.713.5.

6. Zeballos JL, Abidi W, Giménez R, Monforte AJ, Moreno MÁ, Gogorcena Y. Mapping QTLs associated with fruit quality traits in peach [*Prunus persica* (L.) Batsch] using SNP maps. Tree Genet & Genomes. 2016;12:37. doi: 10.1007/s11295-016-0996-9.

7. Salgado Rojas AA. Applying Molecular and Phenotypic Tools to Characterize Flesh Texture and Acidity Traits in the Arkansas Peach Breeding Program and Understanding the Crispy Texture in the Arkansas Blackberry Breeding Program: University of Arkansas, Fayetteville; 2015. Available from: <http://scholarworks.uark.edu/etd/1346/>.
