# Supplementary material for: Identification and characterization of QTLs for fruit quality traits in peach through a multi-family approach
Source: BMC Genomics. 2020 Jul 29;21:522. doi: 10.1186/s12864-020-06927-x (PMC7392839; doi:10.1186/s12864-020-06927-x)
Supplement: Supplementary file 2 — Additional file 2. Supplemental Figures S1 – S7 [file 12864_2020_6927_MOESM2_ESM.docx]

| 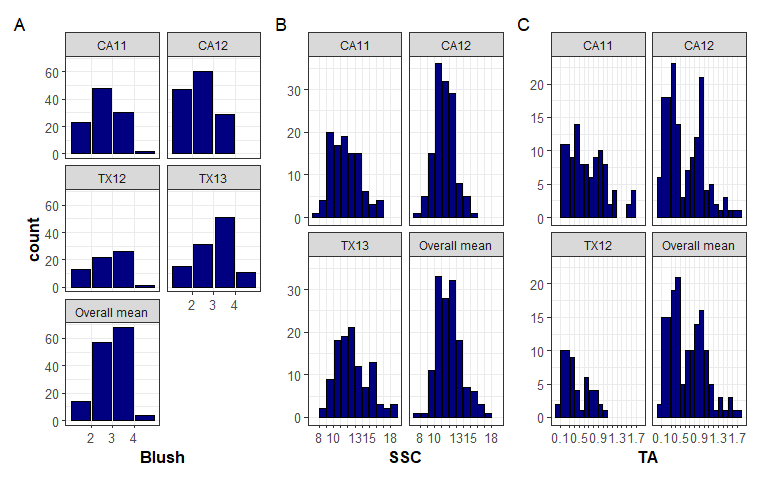 |
| --- |
| Fig. S1. Histograms for blush (A), soluble solids concentration (SSC) (B), and titratable acidity (TA) (C) evaluated in different environments and the overall combined mean.  CA11, CA12 = Fowler, California 2011 and 2012; TX12 = College Station, Texas 2012; Overall mean = mean across environments. |

| 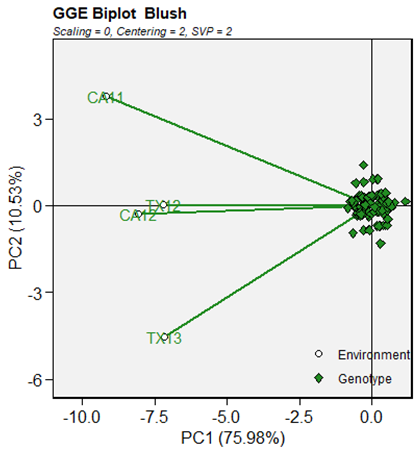 | 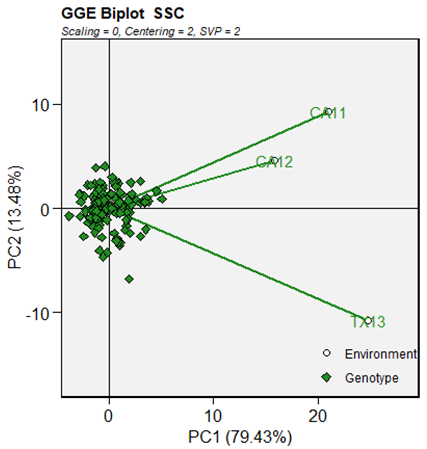 |
| --- | --- |
| 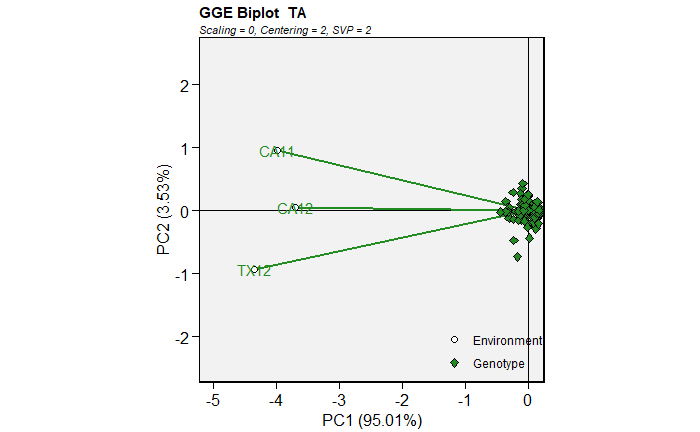 |  |
| Fig. S2. GGE biplots for blush, SSC, and TA showing the relationship between Fowler, CA in 2011 and 2012 and College Station, TX in 2012, and 2013 environments.  TA was not evaluated in TX 2013. | |

| Blush-CA11 | |
| --- | --- |
| 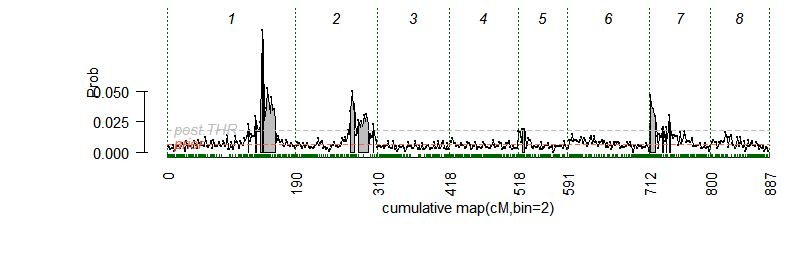 | 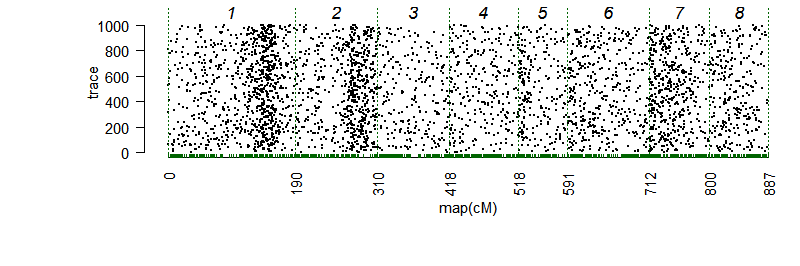 |
| Blush-CA12 | |
| 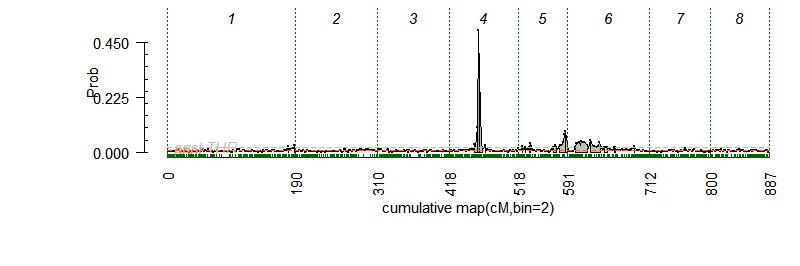 | 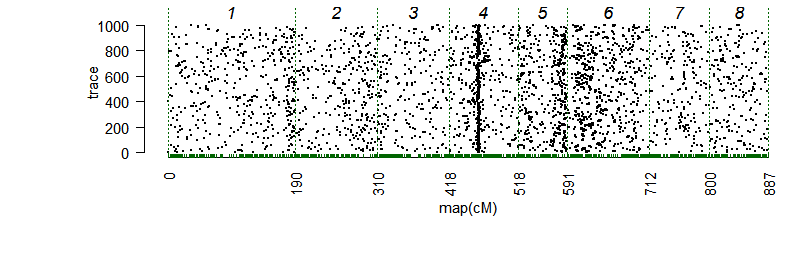 |
| Blush-TX12 | |
| 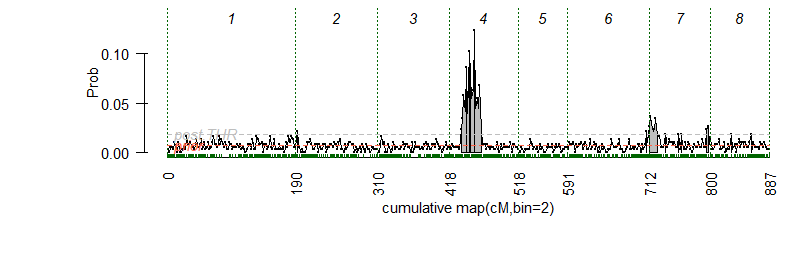 | 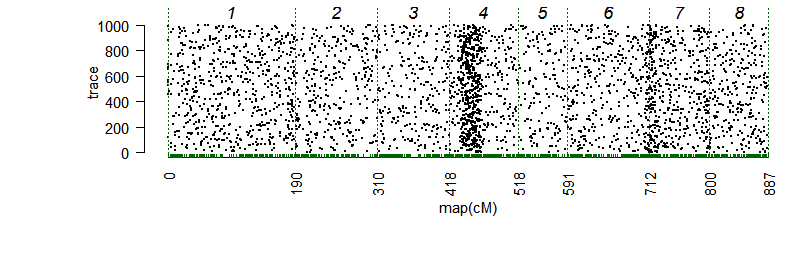 |
| Blush-TX13 | |
| 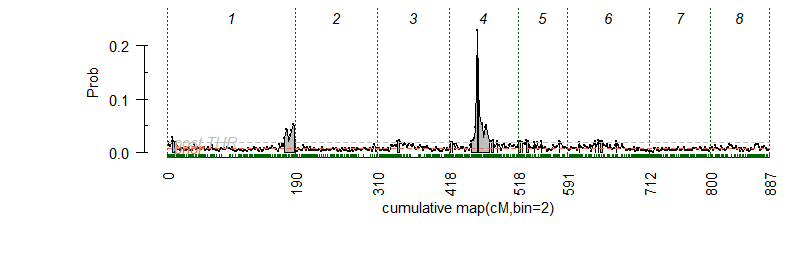 | 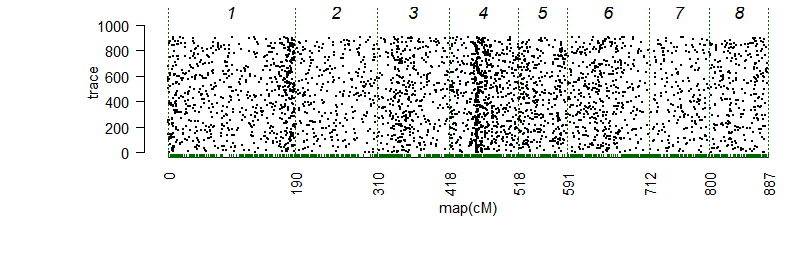 |
| Blush-mean | |
| 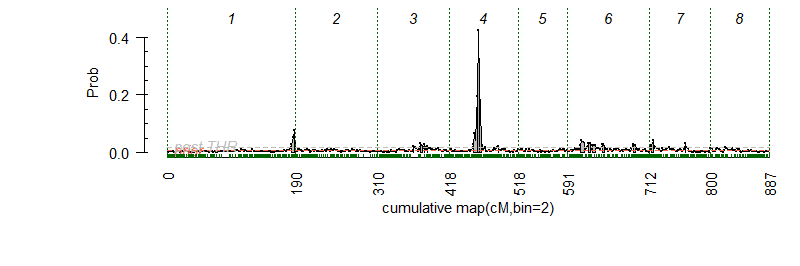 | 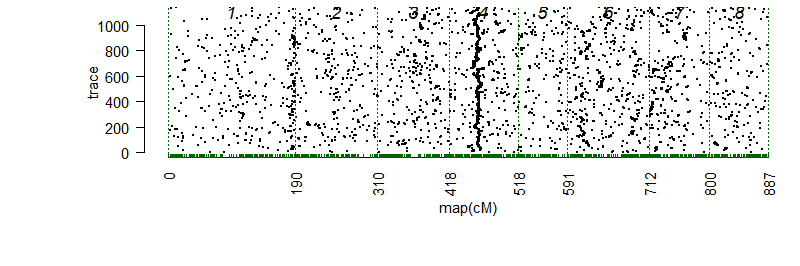 |
| Fig. S3. Posterior positions (left) and trace samples QTL positions (right) based on an additive model performed using Visual FlexQTL software [60] for the blush from four environments (CA11, CA12, TX12, TX13), and the overall combined mean for 143 peach seedlings.  CA11, CA12 = Fowler, California 2011 and 2012; TX12, TX13 = College Station, Texas 2012 and 2013. | |

| SSC-CA11 | |
| --- | --- |
| 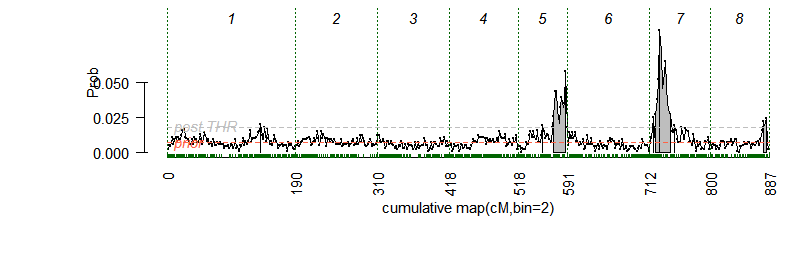 | 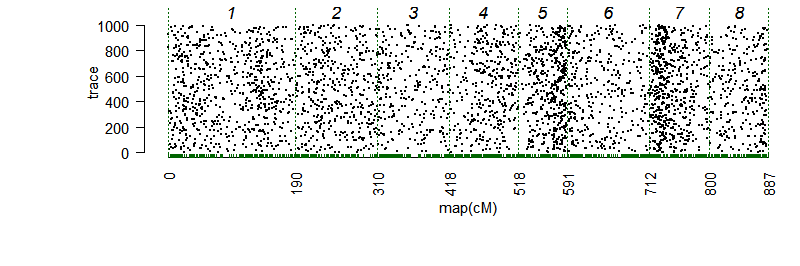 |
| SSC-CA12 | |
| 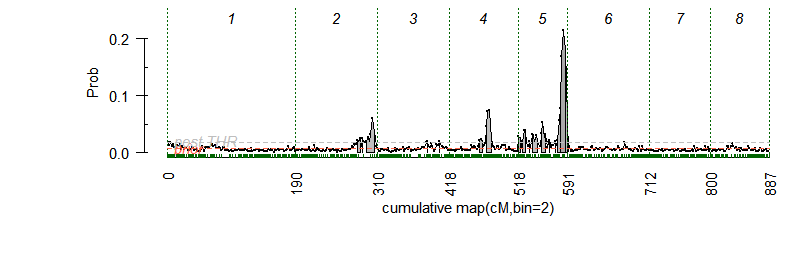 | 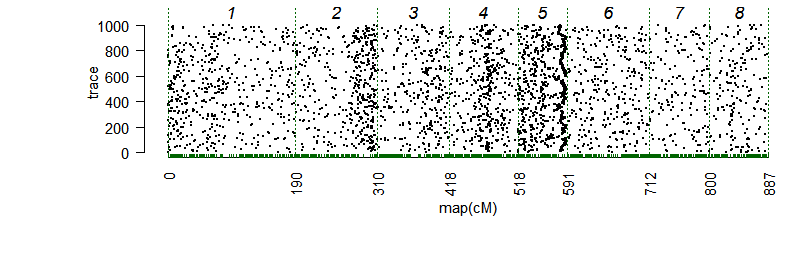 |
| SSC-TX13 | |
| 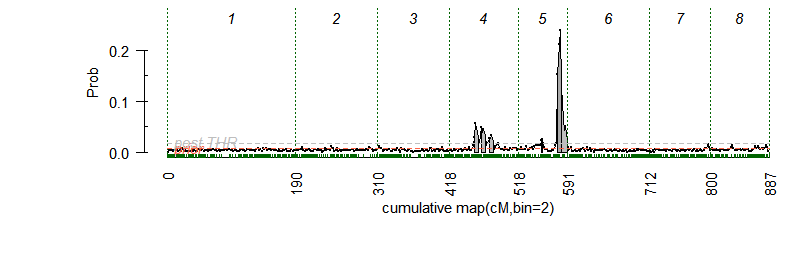 | 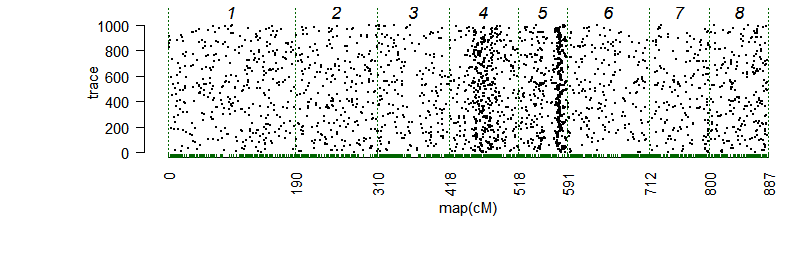 |
| SSC-mean | |
| 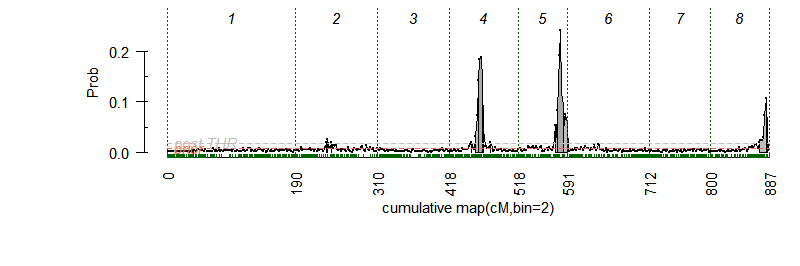 | 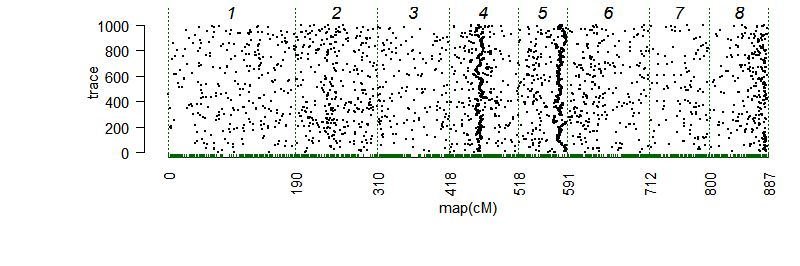 |
| Fig. S4. Posterior positions (left) and Trace samples QTL positions (right) based on an additive model performed using Visual FlexQTL software [60] for the soluble solids concentration (SSC) from three environments (CA11, CA12, TX13), and the overall combined mean for 143 peach seedlings.  CA11, CA12 = Fowler, California 2011 and 2012; TX13 = College Station, Texas 2013 | |

| TA-CA11 | |
| --- | --- |
| 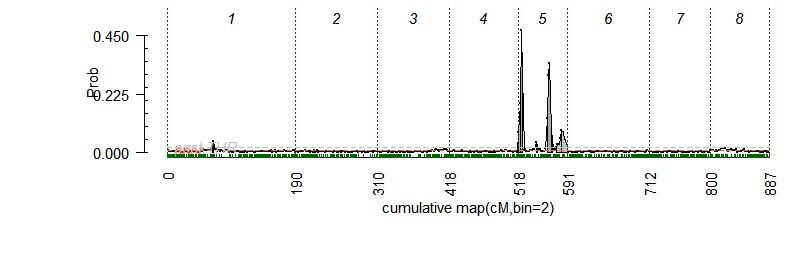 | 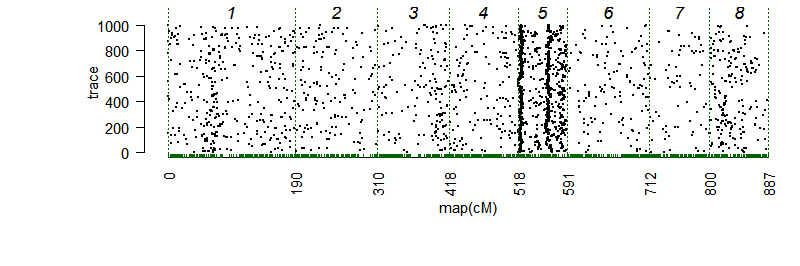 |
| TA-CA12 | |
| 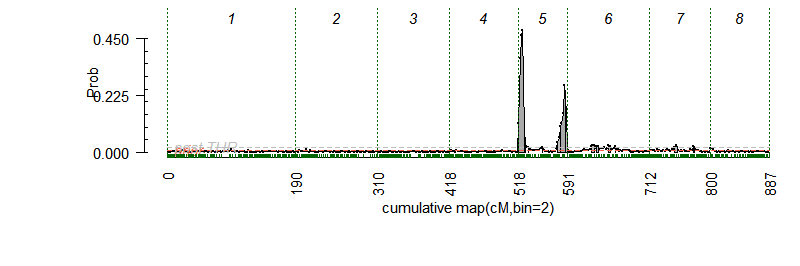 | 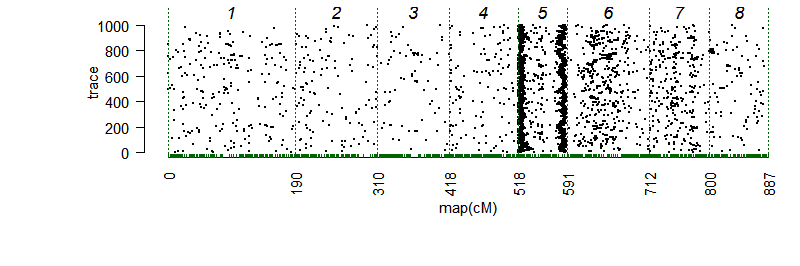 |
| TA-TX12 | |
| 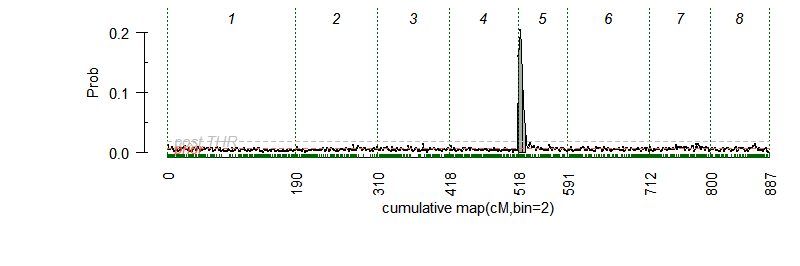 | 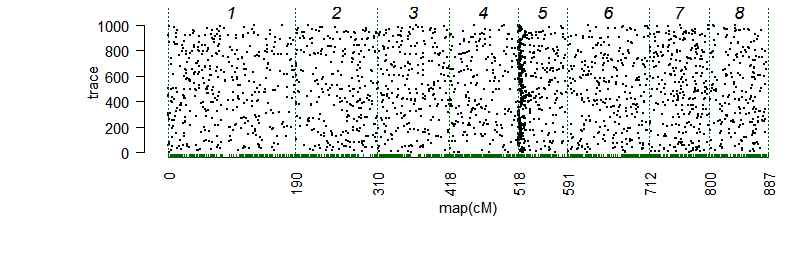 |
| TA-mean | |
| 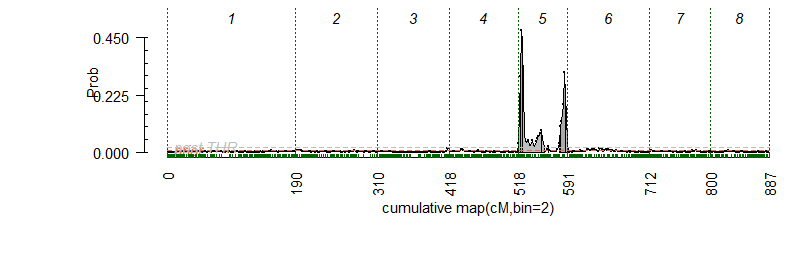 | 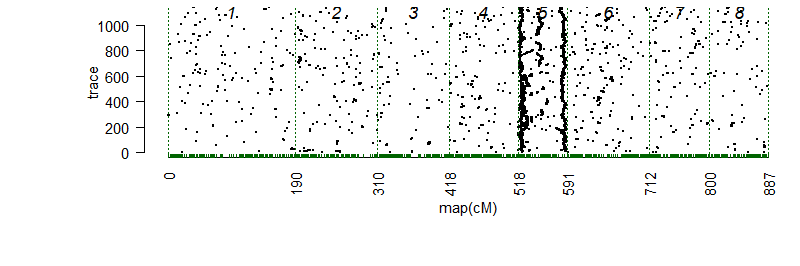 |
| Fig. S5. Posterior positions (left) and trace samples QTL positions (right) based on an additive model performed using Visual FlexQTL software [60] for the titratable acidity (TA) from three environments (CA11, CA12, TX12), and the overall combined mean for 143 peach seedlings.  CA11, CA12 = Fowler, California 2011 and 2012; TX12 = College Station, Texas 2012. | |

|  |
| --- |
| Fig. S6. Correlation plot for titratable acidity (TA) content (%) among environments CA11 and CA12 in peach.  Bubble size represents number of observations.  CA11 = Fowler, California 2011, CA12 = Fowler, California 2012. |

| r = -0.42^**^ |
| --- |
| Fig. S7. Correlation between fruit blush and ripe date (RD).  Blush = blush visually based on % coverage of red blush on skin using 0-5 scale (0 = 0% red coverage, 1 = 1%-20%, 2 = 21%-50%, 3 = 51%-80%, 4 = 81%-99%, 5 = 100%).  Ripe date in Julian days  ^**^ Pearson correlation is significant at the 0.01 level (2-tailed). |
